# Transcriptional dynamics and epigenetic regulation of E and ID protein encoding genes during human T cell development

**DOI:** 10.3389/fimmu.2022.960918

**Published:** 2022-07-28

**Authors:** Juliette Roels, Jolien Van Hulle, Marieke Lavaert, Anna Kuchmiy, Steven Strubbe, Tom Putteman, Bart Vandekerckhove, Georges Leclercq, Filip Van Nieuwerburgh, Lena Boehme, Tom Taghon

**Affiliations:** ^1^Department of Diagnostic Sciences, Ghent University, Ghent, Belgium; ^2^Department of Biomolecular Medicine, Ghent University, Ghent, Belgium; ^3^Cancer Research Institute Ghent (CRIG), Ghent, Belgium; ^4^Laboratory of Pharmaceutical Biotechnology, Ghent University, Ghent, Belgium

**Keywords:** E proteins, ID proteins, T cell development, human, thymocytes, gene regulation, epigenetics, gene regulatory networks

## Abstract

T cells are generated from hematopoietic stem cells through a highly organized developmental process, in which stage-specific molecular events drive maturation towards αβ and γδ T cells. Although many of the mechanisms that control αβ- and γδ-lineage differentiation are shared between human and mouse, important differences have also been observed. Here, we studied the regulatory dynamics of the E and ID protein encoding genes during pediatric human T cell development by evaluating changes in chromatin accessibility, histone modifications and bulk and single cell gene expression. We profiled patterns of ID/E protein activity and identified up- and downstream regulators and targets, respectively. In addition, we compared transcription of E and ID protein encoding genes in human versus mouse to predict both shared and unique activities in these species, and in prenatal versus pediatric human T cell differentiation to identify regulatory changes during development. This analysis showed a putative involvement of TCF3/E2A in the development of γδ T cells. In contrast, in αβ T cell precursors a pivotal pre-TCR-driven population with high ID gene expression and low predicted E protein activity was identified. Finally, in prenatal but not postnatal thymocytes, high HEB/TCF12 levels were found to counteract high ID levels to sustain thymic development. In summary, we uncovered novel insights in the regulation of E and ID proteins on a cross-species and cross-developmental level.

## 1 Introduction

Cellular differentiation is directed by alternating cues for proliferation and differentiation of precursor cells to their final state. In many different cell types E proteins and their inhibitory antagonists ID proteins play an indispensable role in guiding this process. E proteins are basic helix-loop-helix (bHLH) transcription factors that can engage histone modifiers, transcriptional co-activators and DNA binding proteins. As homodimers or heterodimers with other HLH protein family members they bind the six nucleotide CANNTG E box motif in the DNA, which is where their name originates from (1, 2). As such, E proteins can support multiple developmental programs by inducing cell cycle arrest and allowing cellular differentiation (2, 3).

ID proteins, on the other hand, are members of the HLH protein family (4). They can engage with E proteins to inhibit their function by competitive interaction. All ID proteins lack the basic DNA binding domain found in bHLH proteins. Therefore, E-ID dimers cannot bind DNA, which interferes with the E proteins’ transcription factor activity. Generally, the inhibitory interaction of ID with E proteins lifts the cell cycle arrest and promotes cell cycle re-entry at the expense of differentiation, hence their name, Inhibitor of Differentiation (3).

There are three E protein encoding genes, *TCF3* (also known as E2A), *TCF4* (also referred to as E2-2) and *TCF12* (also known as HEB). In addition, TCF3 and TCF12 each have two annotated isoforms that are generated by either alternative splicing (TCF3: E12/E47), or alternative transcription initiation (TCF12: HEBalt/HEBcan), respectively (5). On the other hand, four genes code for ID proteins, namely *ID1* to *ID4.* The level of redundancy between different members of the ID and E protein family is not entirely clear. It is thought that the combined expression level of the different E or ID proteins is a major determinant for differentiation (6–9); however, on top of that, each protein likely has its own unique functions, which can be appreciated by single gene murine knockout experiments (10).

During hematopoiesis, E and ID proteins play an indispensable role at numerous differentiation stages from hematopoietic stem cells (HSCs) to functional myeloid and lymphoid cells. The balance between ID (ID1) and E proteins (E47) can guide HSCs in the direction of myeloid or lymphoid precursors, respectively (11). Similarly, during lymphoid development, the lineage decision between natural killer (NK) and T cells is directed by the ratio of ID to E proteins, with high ID (ID2 and ID3) protein activity favoring NK cell fate (12, 13). In contrast, the fate of Dendritic Cells (DCs) is determined by the activity of different TCF4 isoforms regulating the plasmacytoid DC (pDC) versus conventional DC (cDC) lineage entry (14). The short *TCF4* isoform is expressed in both cDCs and pDCs but is actively repressed by ID2 (under influence of BCL11A) during cDC development. pDCs on the other hand, have specific expression of the long *TCF4* isoform, which is needed for their development (14).

**Figure 1:**
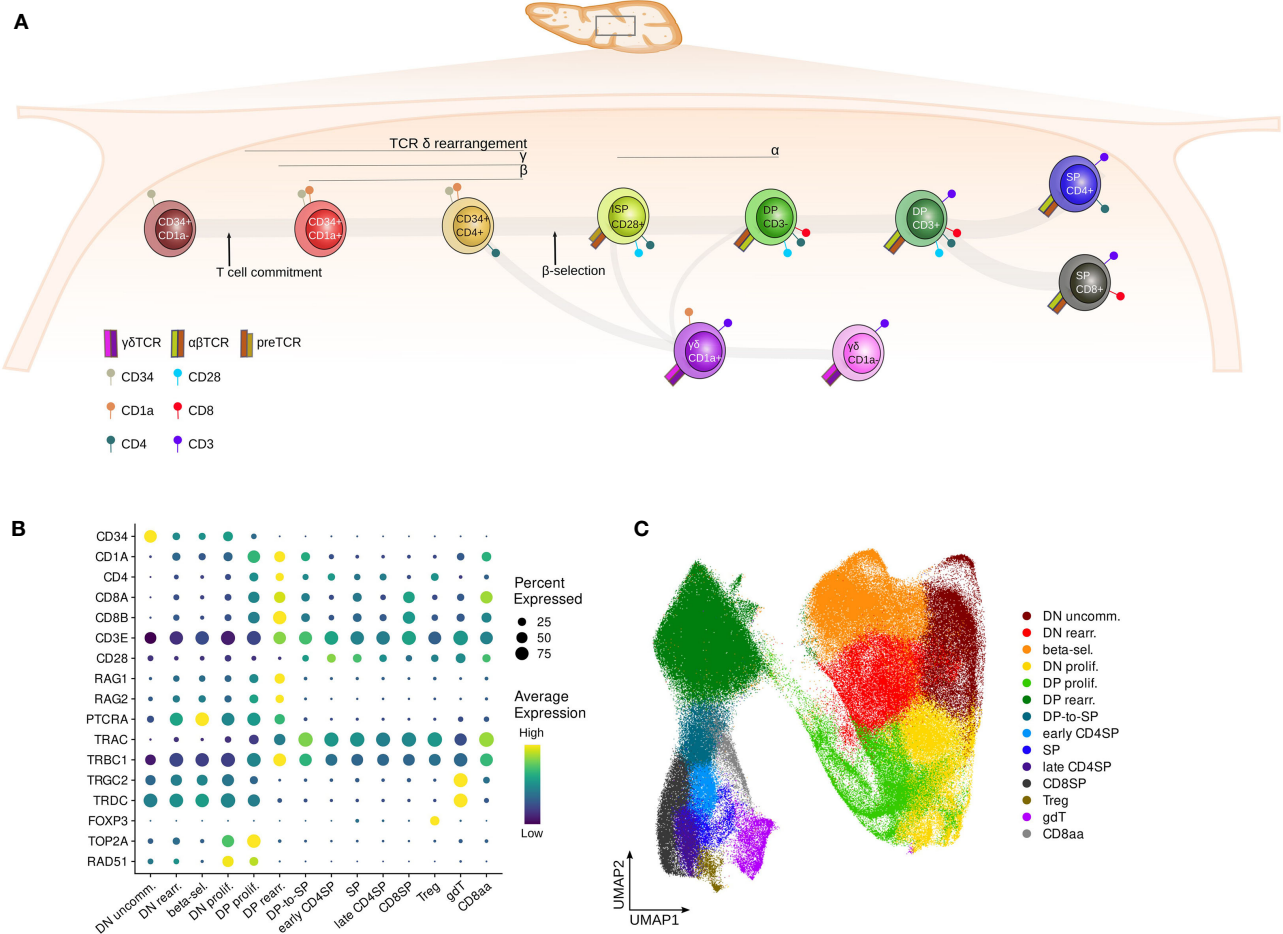
Surface markers distinguish subsequent stages of human thymocyte development. **(A)** Schematic depiction of stages of T cell development in the human thymus. **(B)** Dot plot visualizing pseudo-bulk expression of known thymocyte markers per annotated cluster in the pediatric single cell data set. Non-imputed data was log-normalized, averaged and scaled by gene. **(C)** UMAP visualizing the annotated clusters in the pediatric single cell thymus data set.

T cells develop in the thymus from multipotent lymphoid precursors. During this differentiation process, multiple decision checkpoints exist to generate the wide variety of conventional (αβ) and unconventional (γδ, CD8αα, MAIT, Treg and NK-T) T cells (15, 16). First, bone marrow-derived progenitors gradually differentiate into immature T-lineage specified cells and eventually commit to the T cell fate, excluding potential for other lineages (**Figure 1A**). From here onwards, committed thymocytes start to rearrange their δ, γ and β T cell receptor (TCR) chains in a process known as V(D)J recombination, mediated by the Recombination-Activating Gene (RAG) proteins (17). Successfully rearranged δ and γ chains pair together to form a γδ TCR, which instructs the developing thymocyte to differentiate further into mature γδ T cells, whereas predecessors of αβ T cells require additional selection steps. *TCRB* rearranging T cells form a pre-TCR by combining the β and the invariant pTα chain during a process called β-selection (15). If the pre-TCR signals with adequate intensity, the rearrangement of the α−chain is initiated, which results in progression to the CD4^+^CD8β^+^ double positive (DP) stage of T cell development. DP thymocytes cells undergo negative and subsequently positive selection to ultimately result in mature naïve CD4 or CD8 single positive (SP) αβ T cells. Alternatively, DP thymocytes cells can also give rise to NKT or MAIT cells (18).

During several stages of T cell development E and ID protein driven transcriptional regulation is crucial to ensure proper T cell generation. For instance, the absence of TCF3 partially blocks the earliest stages of thymic differentiation in the mouse (19, 20). TCF3 is an activator of NOTCH1 and of NOTCH1 target genes including *Hes1* and *Dtx1*, thereby priming early thymocyte differentiation (21, 22). Next, TCF3 also activates *Ptcra* and *Rag* expression, which are necessary to initiate TCR rearrangements (22–24). TCF12 is equally essential for T cell development, which is illustrated by *Tcf12*-deficient mice that develop a thymic differentiation arrest, albeit later than *Tcf3*-deficient mice, before the transition to DP thymocytes cells (8). This can in part be explained by TCF12 cooperating with the TCF3 isoform E47 to increase accessibility of the TCR Vβ locus (25). During T cell development, starting from the formation of the pre-TCR, E protein activity is gradually inhibited by ID proteins. This is partially mediated by TCR-mediated induction of *Egr1* expression *via* the RAS–ERK–MAP kinase (MAPK) pathway, which in turn activates *Id3* transcription (26, 27). The γδ TCR is known to give a stronger signal than the pre-TCR (28), which is consistent with high expression of *Id3* in immature γδ T cells (28–31). Moreover, *Id3* expression in γδ T cells remains at higher levels, while a significant decrease in expression can be observed for differentiating αβ thymocytes, further indicating the specificity of ID3 for γδ T cells (32–34). High ID3 activity was recently shown to inhibit *Tcf1* expression in γδ T cells in order to lock in the γδ-lineage fate and effector potential in fetal murine thymocytes (35).

Most of our current knowledge about E and ID protein function during T cell development is based on studies in mice. However, there are some key differences in thymocyte differentiation between mouse and human, especially during the αβ-γδ lineage bifurcation. For instance, both murine and human thymocyte development include an Immature Single Positive (ISP) stage (CD4^+^CD8^-^ in human, CD8^+^CD4^-^ in mice), but while in mice this occurs after β-selection, in human the ISP stage precedes β-selection. Moreover, the order and timing of TCR locus rearrangement differs between the two species, with *TCRD*, *TCRG* and *TCRB* loci rearranging in this chronological order in the human thymus, whereas in mouse V(D)J recombination of the *Tcrb* locus occurs earlier (36). Human thymocytes have also been shown to retain γδ potential throughout a long developmental window since TCRγδ^+^ DP thymocytes can be detected, whereas in wildtype mice, γδ potential is usually extinguished by the time they reach the DP stage (37, 38). Further mechanistic differences between mice and human, such as dependance on Notch signaling, have been established (34).

Similarly, fetal T cell development also differs from postnatal development in several aspects. A quantitative imbalance of thymic output of γδ T cell subsets is observed in prenatal compared to postnatal human T cell development (39, 40). The gene expression dynamics of ID proteins, particularly *ID1* and *ID2*, are also different in fetal compared to postnatal thymocytes as shown by analyses in mice (41). However, whether this difference in expression levels is directly linked to the differences in thymic output is currently unknown. In adult thymocytes, TCF3 does block certain *TCRG* V rearrangements that are specifically recombined in a fetal context (19, 42), which may also indicate a role for E and ID proteins in the balance of γδ T cells before and after birth (43–45).

In this study, we employed bulk and single cell sequencing profiling methods to uncover the regulatory roles of E and ID proteins during human T cell development. We compared our extensive human postnatal thymic data to murine postnatal and human prenatal datasets to gain a better understanding of species-specific and developmental differences, which is of great importance for translational studies. Next, we used gene regulatory network analysis to gain a better understanding of the fine regulatory influence that E and ID protein encoding gene expression has on developing thymocytes. This comprehensive analysis confirmed that many of the findings in mice also hold true in a human context. Nevertheless, we found prominent differences between human and murine expression dynamics of *TCF3*. Furthermore, in human, we found evidence for a regulatory role of TCF3 only after increased accessibility of the *TCRG* locus, which is delayed compared to mouse. Using single cell analysis, we next identified a small cluster of immature γδ T cells that is characterized by *ID3* and *TCF3* expression. In contrast, a cluster of β-selecting cells was identified along the αβ-lineage trajectory that has a very high ID to E protein ratio, likely induced by pre-TCR signaling. Finally, prenatal thymocytes showed an early induction of ID gene expression and stronger TCF12 transcription seems to compensate for this. In conclusion, we here provide a comprehensive analysis of E and ID protein encoding gene activity during thymic differentiation and uncover novel insights into the function of these proteins in different thymic developmental lineages in human.

## 2 Materials and methods

### 2.1 Bulk data analysis

Bulk expression profiling by RNA-seq, chromatin accessibility profiling by ATAC-seq and histone modification profiling (H3K4me3, H3K27ac and H3K27me3) by ChIPmentation was previously generated by our group on developing T cells subsets (46). The IGV Genome Browser was used for visualization of all sequencing tracks. RNA expression counts are shown as transcripts per million reads (TPM).

To identify putative transcription factor binding sites, transcription factor footprinting analysis was performed. Transcription factor footprinting combines information from ChIP-seq derived transcription factor motifs with chromatin accessibility information from ATAC-seq. The presence of a TF prevents the cleavage of DNA, leaving a unique footprint in ATAC-seq reads. This method increases the accuracy of predicting transcription factors’ presence at their binding sites. For footprinting analysis, Bed files generated from ATAC-seq data were used after peak calling with MACS2, as previously described (46, 47). The footprinting analysis was done with the Regulatory Genomics Toolbox (RGT) functions “rgt-HINT” and “rgt-motif analysis matching” (48) using the JASPAR vertebrate motif database (49).

### 2.2 Single cell data generation

#### 2.2.1 Antibodies

CD1a-APC (Biolegend), CD4-PE-Cy7 (Biolegend), CD4-PE (Biolegend), CD8a-FITC (Biolegend), CD8a-APC-Cy7 (Biolegend), CD45-BV510 (BD), CD3-APC (Biolegend)

#### 2.2.2 Cell type enrichment on postnatal thymus samples

Pediatric thymus from children undergoing cardiac surgery was obtained according to and used with the approval of the Medical Ethical Commission of Ghent University Hospital, Belgium. Thymus tissue was cut into small pieces and digested with 1.6 mg/ml collagenase (Gibco, 17104-019) in IMDM medium for 30 min at 37°C to generate a single cell suspension. The reaction was quenched with 10% FBS and the thymocyte suspension was passed through a 70 μm filter to remove undigested tissue. Cells were frozen in FBS containing 10% DMSO and stored in liquid nitrogen until needed. Upon thawing, thymocytes were enriched for cell types of interest (CD34^+^ cells, ISPs, DPs, TCRγδ^+^ thymocytes) using bead-based enrichment/depletion and FACS. To obtain DP thymocytes, cells were labelled with antibodies and FACS sorting was used to obtain equal proportions of CD8α^+^CD4^+^CD3^+^ and CD45^+^CD8α^+^CD4^+^CD3^-^ thymocytes. CD34^+^ cells were obtained through enrichment with CD34 magnetic-activated cell-sorting (MACS) microbeads (Miltenyi, 130-046-703), labelled with anti-CD1a and subsequently FACS sorted to include equal proportions of CD1a^+^ and CD1a^-^ cells. To enrich ISPs, thymocytes were labelled with anti-CD3 (clone OKT3, produced in-house) and anti-glycophorin A (clone 10F7MN, produced in-house) and CD3^+^ and Glycophorin^+^ cells were subsequently depleted using sheep anti-mouse IgG magnetic Dynabeads (Invitrogen). This was followed by FACS sorting for CD3^-^CD8α^-^CD4^+^ thymocytes. To obtain TCRγδ^+^ thymocytes, cells were enriched using anti-γδ TCR Hapten antibodies and anti-Hapten MACS microbeads (Miltenyi, 130-050-701) according to the manufacturer’s instructions and subsequently FACS sorted for TCRγδ^+^CD3^+^.

#### 2.2.3 Library preparation and sequencing

The sorted cells were resuspended in PBS containing 0.04% BSA at a concentration of approximately 1200 cells/μl. 16.5μl cell suspension per sample was loaded onto a Next GEM Chip G (10X Genomics) according to the manufacturer’s instructions and the Chromium Controller was used to generate GEMs. Reverse transcription, amplification and library preparation were carried out using the Next GEM Single Cell 3’ GEM v3.1 kit (10X Genomics) according to the manufacturer’s instructions. Libraries were multiplexed and sequenced to a mean depth of 23.000-54.000 reads/cell.

### 2.3 Single cell data analysis

#### 2.3.1 Preprocessing

Published sequencing data was downloaded from ArrayExpress, GEO and NODE (see Data Availability Statement and Table S1). All fastq files were mapped to the human reference genome GRCh38 using CellRanger version 6.0.1 (10X Genomics). Subsequently, prenatal and pediatric data were analyzed separately. H5 files were loaded into R and analyzed using the Seurat package (50). Cells with over 10% (pediatric data) or 7.5% mitochondrial reads (prenatal data), fewer than 700 reads or expressing fewer than 250 genes were considered to be of low quality and removed from the dataset. The scDblFinder package (51) was used to identify and exclude doublets. In addition, cells with unusually high gene count were removed, with the cutoff varying from >2500 to >6000 genes per cell depending on the sequencing depth of the respective library. Finally, genes expressed in fewer than 10 cells across the entire dataset were removed as non-informative.

Gene expression was log-normalized and the 2000 most variable genes (HVGs) were identified using Seurat. To correct for cell cycle-dependent effects but preserve information about proliferative vs. quiescent cell states, cell cycle scoring was conducted using the G2/M and S phase marker genes provided in the Seurat package and the difference between G2M and S scores was regressed out. Moreover, differences in sequencing depth between samples were regressed out and data was scaled and centered.

#### 2.3.2 Dimensionality reduction, batch correction and clustering

PCA was performed on the scaled HVGs. To reduce batch effects between samples, MNN correction was applied to the PCA matrix *via* the reducedMNN function from the Batchelor package (52). For this step, every library was considered as a separate batch and the merge order was manually specified to guarantee the largest possible overlap in cell types between subsequently merged libraries. The corrected PCA was used to generate an SNN graph (k=50), which was then used to conduct Louvain clustering with an initial resolution of 0.3. Large clusters were further subclustered with a resolution of 0.1-0.8 to identify additional subpopulations of interest. UMAP was used to visualize the results and known marker genes for distinct stages of thymocyte development were used to annotate the clusters (**Figure 1B**). Clusters with comparable expression of marker genes were merged to obtain the larger annotated clusters used for downstream analyses (**Figure 1C**). Non-relevant clusters, such as dendritic cells, B cells, stromal cells and NK cells, were removed from the dataset prior to downstream analyses.

#### 2.3.3 DGE, imputation and cell scoring

Differential gene expression analysis for clusters of interest was carried out in a one-vs-all manner on the normalized data *via* the FindMarkers function from the Seurat package using a Wilcoxon Rank Sum test and Bonferroni correction. Prior to visualization, Markov affinity-based graph imputation of cells (MAGIC) (53) was used to denoise the data and impute dropout values.

The UCell package (54) was used to perform cell scoring. To establish the E:ID score, *TCF3*, *TCF4* and *TCF12* were given positive weights while *ID1*, *ID2* and *ID3* carried negative weights. For Notch scoring the following genes were considered indicators of Notch signaling activity: *TCF7*, *HES1*, *HES5*, *HEY1*, *DTX1*, *NOTCH1*, *NOTCH3*, *IL7R*, *CD7*, *PTCRA*, *MYC*, *CCND1*, *NRARP* and *TCF3*.

#### 2.3.4 Pseudotime analysis

The destiny package (55) was used to establish a diffusion map based on the first 20 principal components. Subsequently, the first diffusion component was used as pseudotime measure. Proliferating cells showed inconsistent clustering and were therefore removed from the pseudotime ordering; moreover, only αβ-lineage cells were included in the analysis. The tradeseq package (56) was used to fit a generalized additive model (GAM) on the cell pseudotimes and to determine smoothed gene expression values. Data was scaled and plotted using pheatmap (57).

#### 2.3.5 Gene Regulatory Network (GRN) analysis

GRN analysis was conducted using pySCENIC (58) and SIGNET (59). Due to the compute-intensive nature of the two pipelines the dataset was downsampled to a representative subset of 50.000 and 10.000 cells, respectively. A list of human transcription factors as well as motif ranking databases (mc9nr hg38 500bpUp100Dw and TSS+/-10kbp) were obtained from the online resources provided by the Aerts lab.

In line with the recommended pySCENIC workflow, the GRNBoost2 algorithm (60) was used to determine co-expression modules between transcription factors and potential targets. Subsequently, regulon prediction was carried out using cisTarget based on HGNC motif annotations and motif ranking databases. Finally, the regulon activity per cell was determined *via* enrichment scoring for the regulon target genes using AUCell (61).

For detection of transcription factor-target co-expression modules with SIGNET the same list of transcription factors was supplied as for pySCENIC. RcisTarget (61) was used to prune the modules based on motif rankings and HGNC annotations.

#### 2.3.6 Automated cell type annotation of prenatal data

The singleR package (62) was used to carry out automated annotation of cell types in the prenatal dataset. For this purpose, a pseudobulk gene expression reference was generated from the pediatric single cell dataset. SingleR was then used to infer labels for individual cells based on similarity to the gene expression signature of the annotated clusters in the pediatric data.

## 3 Results

### 3.1 Expression of E and ID protein encoding genes throughout human thymocyte development

To obtain a better understanding of the activity of E and ID proteins during human T cell development, we made use of bulk RNA-seq, ATAC-seq and ChIPmentation data for distinct stages of human thymocyte development as described earlier (46) (**Figure 1A**). In addition, we compiled a comprehensive scRNA-seq dataset from multiple different sources (63–65) including several new libraries (**Figure S1A, Table S1**), incorporating approximately 280.000 thymocytes from 13 pediatric donors between the ages of 9 days and 13 years. Sufficient coverage of rare developmental stages was achieved through enrichment for specific cell types prior to library preparation (**Figures S1B** and **Table S1**). UMAP-based dimensionality reduction, unsupervised clustering (**Figure S1C**) and manual annotation of the data based on known cell type markers (**Figures 1B** and **S1D, E**) was carried out. In this process, subclusters with comparable marker gene expression were merged to form larger annotated cell populations (**Figure 1C**). This confirmed that the single cell dataset spans thymocytes across all developmental stages, from the most immature precursors to the fully differentiated naïve T cells (**Figure 1C**).

To establish gene expression trends along thymocyte differentiation, we evaluated the transcript levels of the genes that encode each of the E and ID proteins in both the bulk samples, and in the continuum of the single cell dataset.

**Figure 2:**
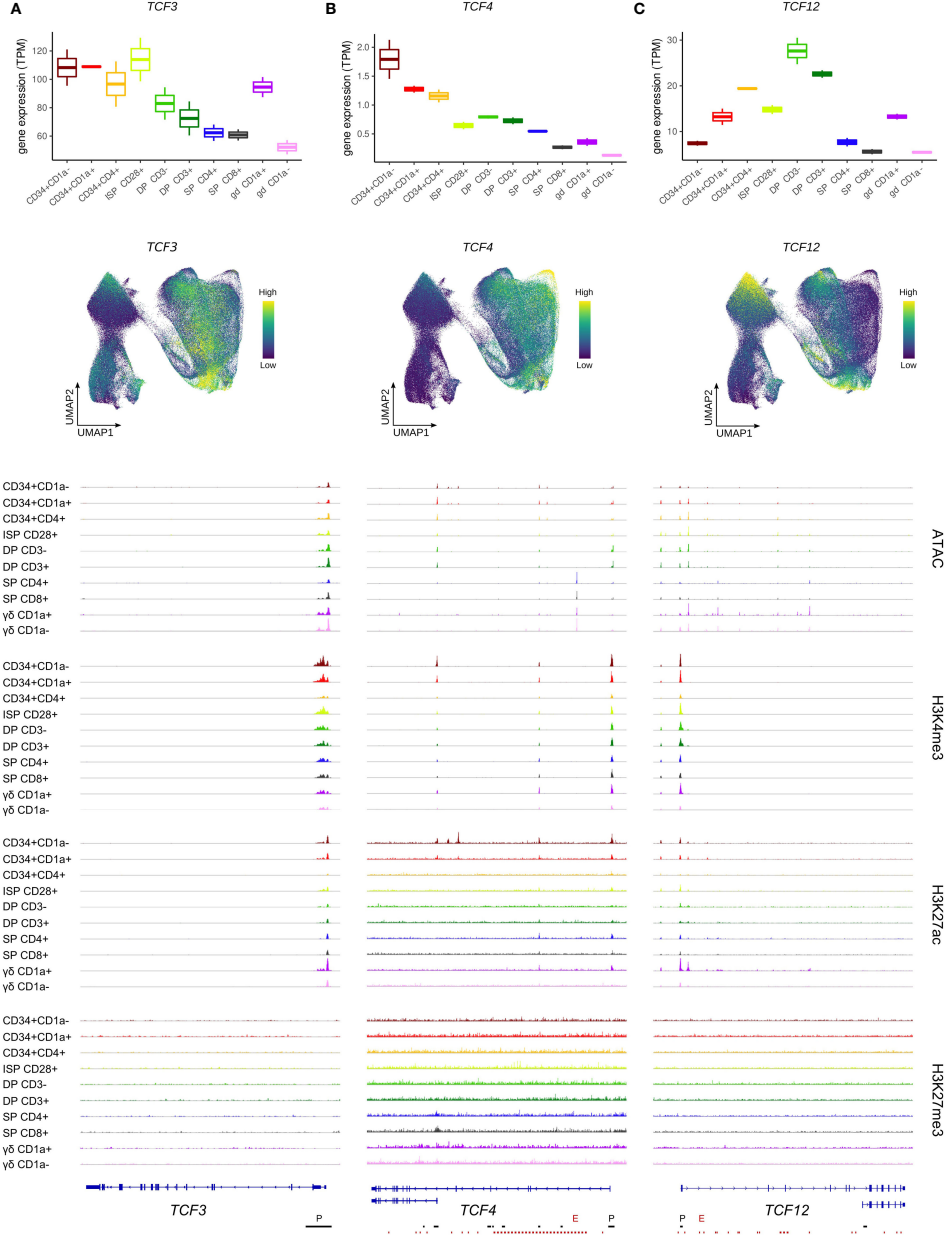
Expression of E protein encoding genes throughout human thymocyte development. **(A)** Transcript levels of *TCF3* according to bulk RNA-seq (top) and single cell data (middle), and epigenetic profile at the *TCF3* gene locus. **(B)** Transcript levels of *TCF4* according to bulk RNA-seq (top) and single cell data (middle), and epigenetic profile at the *TCF4* gene locus. Long and short isoform of TCF4 are shown. **(C)** Transcript levels of *TCF12* according to bulk RNA-seq (top) and single cell data (middle), and epigenetic profile at the *TCF12* gene locus. Long (HEBcan) and short isoform (HEBalt) of TCF12 are shown. Locations of promoters (P) and enhancers (E) were retrieved from the Ensembl Regulatory Build and are indicated below the gene structure. UMAP visualizations were generated using imputed data.

Analysis of *TCF3* RNA levels revealed high expression in immature thymocytes up until the earliest lineage-specific stages of αβ and γδ T cell development (β-selected ISP CD28^+^ and TCRγδ ^+^CD1a^+^ cells, respectively), followed by a gradual downregulation in both lineages with ongoing maturation (**Figure 2A**, top+middle). Even though *TCF3* gene expression was reduced in more mature thymocytes, active promoter marks (H3K4me3 and H3K27ac) and a complete absence of repressive chromatin modifications (H3K27me3) were detected at the *TCF3* locus throughout thymocyte development, supporting sustained *TCF3* transcription (**Figure 2A**, bottom). Furthermore, we detected the expression of both TCF3 isoforms *E12* and *E47* at comparable levels in the bulk RNA-seq dataset (data not shown).

Similar to *TCF3*, *TCF4* transcript levels were found to follow a downward trend as T cell development progressed (**Figure 2B**, top+middle). However, *TCF4* transcription already decreased early on, at the T cell commitment stage (CD34^+^CD1a^+^), and the immature αβ/γδ-lineage cells showed a substantial reduction in *TCF4* RNA levels compared to preceding stages. This suggests a swift shutdown of *TCF4* transcription in differentiating thymocytes, in contrast to *TCF3* expression, which is maintained throughout a wider developmental window. Downregulation of *TCF4* was associated with a moderate reduction of H3K27ac and H3K4me3 at the gene promoter of the long *TCF4* isoform (**Figure 2B**, bottom). However, the promoter of the short *TCF4* isoform displayed profound H3K27ac marks in the earliest developmental stages but was completely shut down by the CD34^+^CD4^+^ stage. Ultimately, for both isoforms, a decrease in chromatin accessibility after the DP stage was observed, consistent with the drop in transcriptional activity (**Figure 2B**, bottom).

The third E protein encoding gene, *TCF12*, exhibited a very different expression pattern with low RNA levels in uncommitted thymocytes and an initial peak around the putative αβ/γδ bifurcation point (CD34^+^CD4^+^) (**Figure 2C**, top+middle). In cells of the γδ-lineage, *TCF12* transcription was subsequently reduced, while αβ-lineage cells were found to experience a second window of strong *TCF12* expression at the DP stage, followed by rapid downregulation at the DP-SP transition. TCF12/HEB has two known isoforms, HEBcan and HEBalt, both originating from alternative transcript initiation (5). In our dataset, there was no evidence for a distinctly active alternative start site for *HEBalt* in developing human thymocytes. Indeed, a complete absence of open chromatin or active promoter methylation was observed at this site (**Figure 2C**, bottom) and expression of the N-terminal HEBalt-specific exon was not detected. Therefore, *HEBalt* transcripts are presumably only very lowly expressed, if at all, in our human thymic dataset.

In contrast to the *HEBalt* promoter, the promoter region of *HEBcan* did exhibit H3K4me3 and K3K27ac histone marks, as well as open chromatin. This was already evident in the most immature thymocytes, thus preceding the higher transcription levels, which suggests that the most immature thymocytes are primed for *TCF12* upregulation (**Figure 2C**, bottom). Shutdown of *TCF12* expression in the αβ-lineage was accompanied by chromatin closure and loss of H3K27ac in SP thymocytes. In contrast, in γδ-lineage thymocytes permissive chromatin marks were lost in mature CD1a^-^ cells, but chromatin accessibility was maintained, suggesting a different mechanism of transcriptional downregulation in these cells.

**Figure 3:**
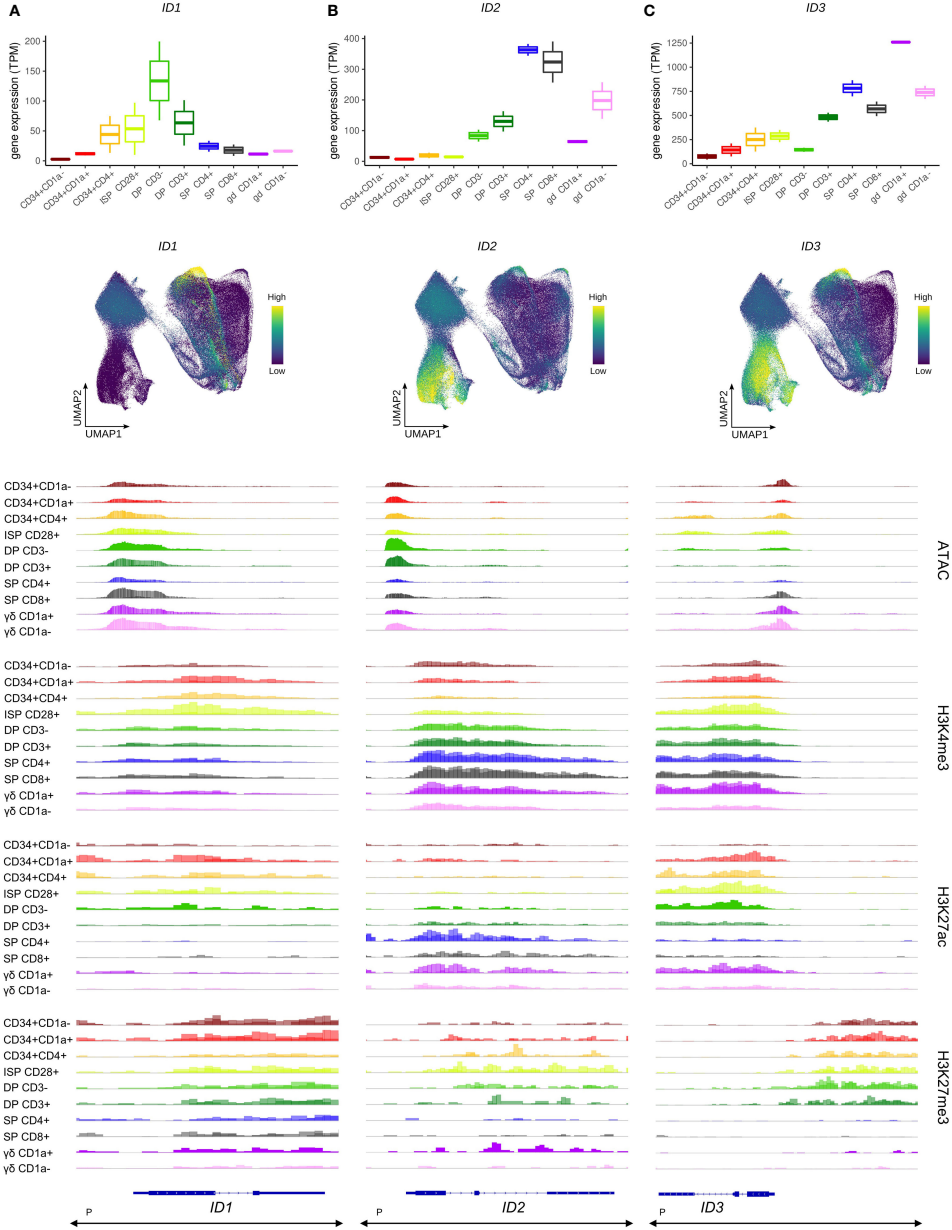
Transcription of ID protein encoding genes in developing human thymocytes. **(A)** Transcript levels of *ID1* according to bulk RNA-seq (top) and single cell data (middle), and epigenetic profile at the *ID1* gene locus. **(B)** Transcript levels of *ID2* according to bulk RNA-seq (top) and single cell data (middle), and epigenetic profile at the *ID2* gene locus. **(C)** Transcript levels of *ID3* according to bulk RNA-seq (top) and single cell data (middle), and epigenetic profile at the *ID3* gene locus. Locations of promoters (P) for all three genes was retrieved from the Ensembl Regulatory Build and was found to span the entire locus as indicated below the gene structure. UMAP visualizations were generated using imputed data.

Since E protein activity is crucially controlled through inhibitory dimerization with ID proteins (66), we also assessed ID transcript levels throughout thymocyte development. *ID1* gene expression exhibited a rapid increase, followed by a steep decline, with the highest levels detected in CD3^-^ DP thymocytes, whereas few *ID1* transcripts were identified in the preceding immature stages or the more mature TCRαβ^+^ SP and TCRγδ^+^ thymocytes (**Figure 3A**, top). This pattern resembles that of *TCF12*, but comparison of expression at the single cell level on UMAP showed that there was surprisingly little overlap in cells expressing high levels of *ID1* or *TCF12* (**Figure 3A**, middle, and **Figure S2A**). Indeed, *ID1* expression was found to be rather heterogeneous especially in rearranging and β-selecting thymocytes, with some cells exhibiting strong *ID1* expression whereas other cells at the same developmental stage showed very low *ID1* transcript levels. This suggests that bulk expression profiles indeed do not entirely reflect the fine-grained dynamics of *ID1* expression throughout early differentiation. Remarkably, the *ID1* locus was marked by both repressive and activating histone modifications (H3K27me3 and H3K27ac) (**Figure 3A**, bottom). This suggests the presence of poised regulatory elements that can rapidly and temporarily switch to active promoters/enhancers over the course of development, which is consistent with the expression dynamics observed in the single cell data.

In the bulk data, *ID2* transcription was not detected in the most immature stages of human T cell development (**Figure 3B**, top), although the single cell data suggested two small subsets of cells expressing ID2 in the immature DN and β-selection clusters (**Figure 3B**, middle). Widespread induction of *ID2* was observed in both the αβ- and γδ-lineage committed cells and reached peak levels in the most mature SP and TCRγδ^+^ cells (**Figure 3B**, top+middle). The increased expression in later stages of thymic development was accompanied by higher H3K4me3 and H3K27ac levels (**Figure 3B**, bottom). Combined, this suggests a role for ID2 in the late stages of T cell development or maybe even only in mature cells, with limited function in the early differentiation steps.

*ID3* gene expression followed a similar pattern, with low RNA levels throughout the most immature stages and a progressive upregulation during αβ-lineage differentiation in DP and SP thymocytes (**Figure 3C**, top), although induction seemed to occur slightly earlier than that of *ID2* as visible in the UMAP plots (**Figures 3B, C**, middle). In contrast, in the γδ-lineage a striking increase in ID3 transcripts was evident in immature TCRγδ^+^ cells, which is consistent with reports of ID3 being an important regulator of murine γδ T cell development (29, 67). The strong initial upregulation of *ID3* in immature CD1a^+^ γδ T cells was followed by a reduction during further γδ-lineage maturation to levels comparable with those in SP TCRαβ^+^ thymocytes (**Figure 3C**, top). In addition, the single cell data suggested a drop in *ID3* levels in more mature SP thymocytes, which was not discernible from the bulk expression profiles (**Figure 3C**, middle). In disagreement with its expression pattern, *ID3* was found to exhibit high levels of H3K27ac at the transcription start site and in the gene body in immature thymocytes, which were extinguished by the SP stage (**Figure 3C**, bottom). H3K4me3 marks were also found in the gene body throughout most developmental stages and therefore cannot explain the transcriptional upregulation of the *ID3* gene in immature TCRγδ^+^ cells and SP thymocytes. However, in these *ID3*^high^ cell types a prevalent H3K27me3 site immediately upstream of *ID3* was remarkably depleted of this histone modification, while it had persistent methylation from the most immature stages up until the DP-SP transition (**Figure 3C**, bottom). In addition, chromatin accessibility at the *ID3* transcription start site was increased in cells of the γδ lineage. Thus, the involvement of both H3K27ac and H3K27me3 as well as chromatin opening in cell type-specific regulation of *ID3* expression point again to a very complex regulatory mechanism of *ID* gene expression during thymic development.

Finally, no noticeable *ID4* expression was detected in any of the thymocyte stages, which is consistent with previous reports (68) (**Figure S2B**).

### 3.2 E and ID protein encoding gene expression in human and murine thymic development

Thymic expression of E and ID protein encoding genes in the mouse has been studied in detail (7, 25, 69–73) and transcript levels at distinct stages have been mapped by the Immunological Genome Project Consortium (74). We made use of this resource to perform an inter-species comparison of gene expression trends during thymocyte development. Of note, a direct and accurate stage-by-stage comparison between mouse and human is difficult since some developmental stages do not have matching phenotypic markers in both species, especially the most immature thymocyte stages.

In general, highly similar trends were observed for the expression of most E and ID genes in human and mouse thymocytes (**Figure S3**). *ID2* and *ID3* displayed the same late upregulation in both species, with peak expression in the αβ-lineage SP and the γδ T cell stages, respectively (**Figure S3**). Likewise, high initial levels of *TCF4* expression and its subsequent downregulation were observed in murine and human cells. The previously described bimodal expression profile of *TCF12* with peaks around the human β-selection checkpoint and in DP thymocytes was also mirrored in the mouse.

*TCF3* expression peaked in ISP thymocytes in both species, even though these represent different developmental stages in both species, but in human thymocytes this was preceded by consistently high expression levels, whereas murine thymocytes showed only a gradual *Tcf3* upregulation with low levels at the DN1 stage (**Figure S3**). Some differences were also observed in the transcription profile of *ID1*, which displayed peak expression in DP thymocytes of both human and mouse but seemed to fluctuate in mouse DN thymocytes (**Figure S3**). This variability might be caused by transient or heterogeneous upregulation of *Id1* throughout the DN stage, as previously noted for the human single cell dataset (**Figure 3A**, middle). Alternatively, variable *Id1* expression may be attributed to the overall low levels of *Id1* in mouse thymocytes, especially when compared to those of *Id2* and *Id3*. This raises questions about the biological relevance of *Id1* expression in the mouse thymus, whereas *ID1* levels in human thymocytes are moderately high and may therefore reflect an actual functional role for ID1 in human T cell development.

Despite the overall similarities in the transcriptional dynamics of E and ID protein encoding genes in human and murine thymocytes, the few observed discrepancies should be considered when attempting to model human T cell development in a mouse system. Especially the differences in the *TCF3* expression profiles between the two species suggest that many findings regarding TCF3 functions in early thymocyte differentiation in mouse might need caution when translating to human.

### 3.3 E and ID protein encoding genes during initial lineage decisions in the thymus

To gain deeper insight into the biological significance of the expression of E and ID protein encoding genes in differentiating thymocytes, we carried out trajectory analysis and gene regulatory network (GRN) prediction on the single cell data, and transcription factor footprinting analysis on the bulk RNA-seq and ATAC-seq datasets.

**Figure 4:**
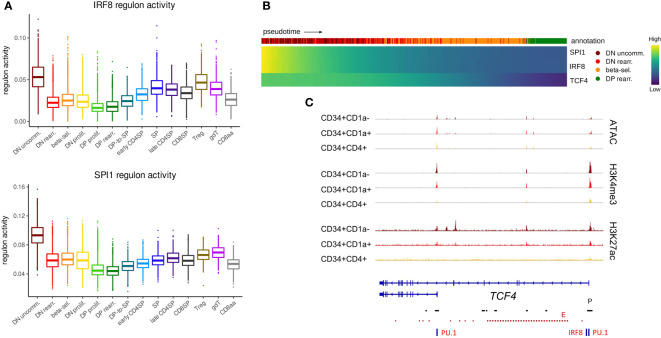
TCF4, IRF8 and SPI1 are predicted to form a regulatory network in uncommitted DN thymocytes. **(A)** Activity of IRF8 and PU.1 regulons per cluster as predicted by running pySCENIC on the single cell data set. **(B)** Heatmap showing gene expression along differentiation pseudotime in immature thymocytes. Smoothed gene expression was determined based on a generalized additive model fitted on the cell pseudotimes, cells in pseudotime window of interest were selected and expression was scaled by gene prior to visualization. **(C)** Genome browser view of ATAC, H3K4me3, H3K27ac and PU.1/IRF8 motifs at the *TCF4* locus. Locations of promoters (P) and enhancers (E) were retrieved from the Ensembl Regulatory Build and are indicated below the gene structure.

Given the important role for E and ID proteins during lineage decisions, we assessed E and ID gene regulation at the earliest stages of T cell development, during which cells can still branch off towards other hematopoietic lineages. Indeed, at the most immature stage, which represents a subset of CD34^+^CD1a^-^ cells, thymocytes have not yet fully committed to the T-lineage and still have the potential to give rise to other non-T cell types, including DCs (63, 75). Our gene expression analysis indicated high levels of *TCF4* RNA in these immature cells, but continuous downregulation in T-committed thymocytes (**Figure 2B**, top), suggesting a potential role very early on in thymocyte differentiation. GRN analysis on the single cell dataset identified two regulons with exceptionally high activity in the most immature thymocytes, which was quickly extinguished in subsequent stages (**Figure 4A**). Interestingly, both regulons included *TCF4* as a target gene and were predicted to be driven by *IRF8* and *SPI1* (encoding PU.1). Expression of these two transcriptional regulators was indeed found to be high in immature thymocytes and preceded that of *TCF4* (**Figure 4B**). Moreover, PU.1 and IRF8 footprints were detected in the open, active chromatin regions at the *TCF4* regulatory elements in CD34^+^ thymocytes (**Figure 4C**). TCF4, PU.1 and IRF8 are all known to be crucial transcription factors for DC development (76–78) but a previous study seems to place TCF4 upstream of PU.1 and IRF8 (79). In accordance with this, E protein motifs were indeed also detected at the *SPI1* and *IRF8* loci (data not shown). However, our regulon prediction results and temporal order of *TCF4*, *SPI1* and *IRF8* expression in immature thymocytes raise the possibility of TCF4 not (just) as regulator but also as target of *SPI1*/PU.1 and IRF8. In addition, they strongly suggest that *TCF4* expression in immature thymocytes might reflect a more prominent role in supporting DC compared to T cell development.

The expression of a short *TCF4* isoform has previously been described in cDCs and pDCs as well as other cell types, whereas the long *TCF4* isoform seems to be exclusively expressed in pDCs (14). Therefore, we assessed the footprint analyses of the promoters of the long and short *TCF4* isoform separately. The long *TCF4* isoform, which displayed more stable chromatin accessibility, was shown to be driven by both IRF8 and PU.1 (**Figure 4C**). In contrast, for the short isoform we only found evidence for binding of PU.1 but not IRF8. Interestingly, previous research identified PU.1 as a repressor of pDC fate within the DC-lineage (80). Therefore, we hypothesize a role for the interplay between PU.1 and the long *TCF4* isoform in guiding immature DCs (or CD34^+^CD1a^-^ unspecified thymocytes) to the pDC fate. Moreover, the differing dynamics of epigenetic changes at the individual promoters suggest divergent expression windows and upstream regulators for both *TCF4* isoforms, although the consequences of this remain to be established.

**Figure 5:**
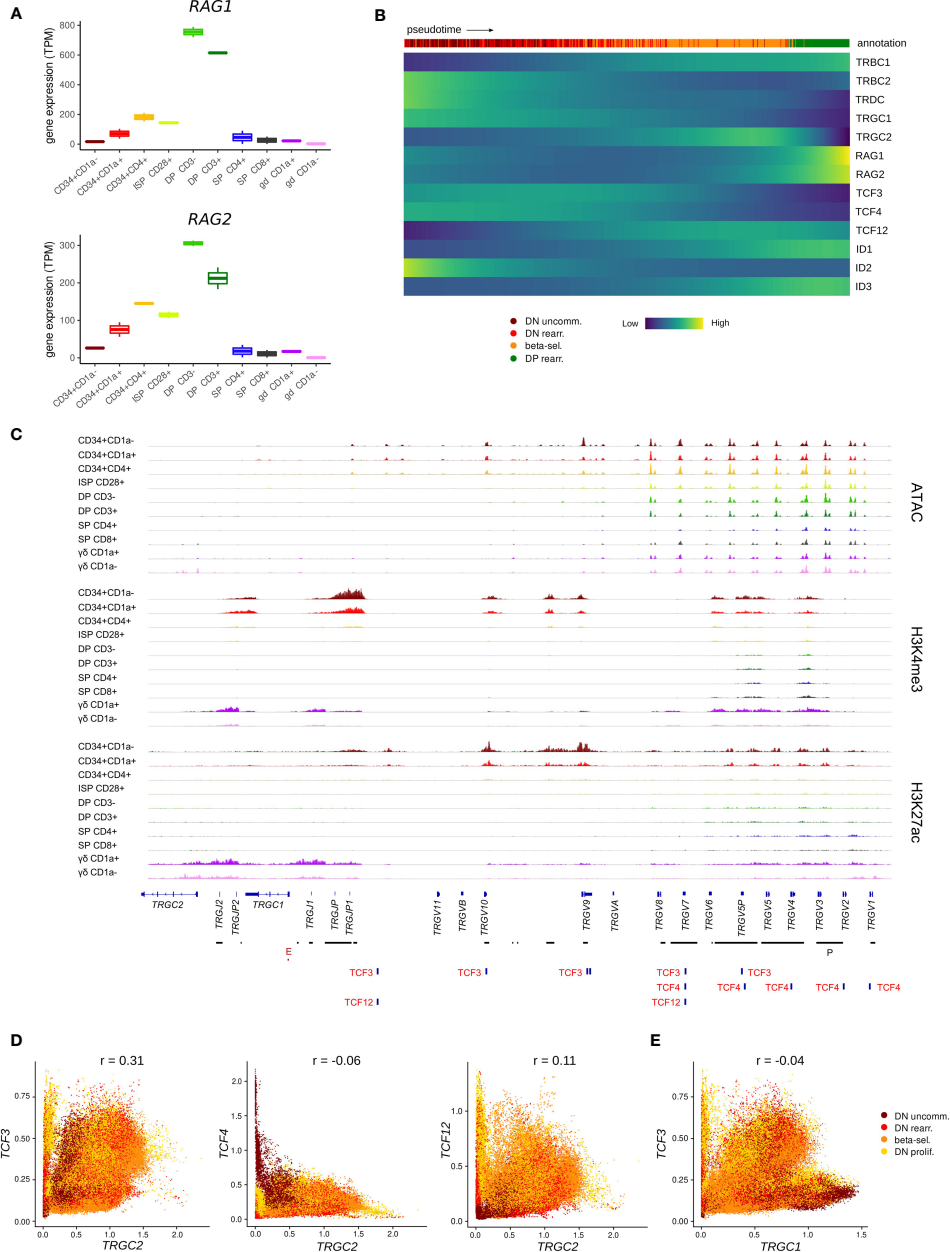
*TCF3* expression is positively correlated with transcription of *TRGC2*. **(A)** Expression of *RAG1* and *RAG2* at different stages of thymocyte development according to bulk RNA-seq data. **(B)** Heatmap showing gene expression along differentiation pseudotime in immature thymocytes. Smoothed gene expression was determined based on a generalized additive model fitted on the cell pseudotimes, cells in pseudotime window of interest were selected and expression was scaled by gene prior to visualization. **(C)** Genome browser view of ATAC, H3K4me3, H3K27ac and E protein motifs at the *TCRG* locus. Locations of promoters (P) and enhancers (E) were retrieved from the Ensembl Regulatory Build and are indicated below the gene structure. **(D)** Scatter plot for imputed transcript levels of E protein encoding genes and *TRGC2* in immature thymocytes. Cells are colored by cluster and Pearson correlation coefficient is shown. **(E)** Scatter plot for imputed transcript levels of *TCF3* and *TRGC1* in immature thymocytes. Cells are colored by cluster and Pearson correlation coefficient is shown.

Once committed to the T-lineage, thymocytes are still bipotent and can adopt either the αβ or γδ T-cell fate, depending on the TCR that they assemble and the signals they receive. The expression patterns of E and ID protein encoding genes suggested particularly high E but low ID encoding transcript levels throughout the first stages of thymocyte development. This indicates potentially strong E protein activity in this phase, which prompted us to investigate the possible consequences. To develop into functional T cells, thymocytes undergo V(D)J recombination to be able to produce a wide range of TCRs with different specificities. For the *TCRD*, *TCRG* and *TCRB* loci, this rearrangement takes place during the immature stages that precede β-selection and the DP stage. Several studies in mice have implicated TCF3 in *Tcrg* locus accessibility and consequently in initiation and regulation of V(D)J recombination of this gene (72, 81) but known differences exist between human and mice in the order and coordination of TCR locus rearrangements (36). Therefore, we explored the possibility of TCF3 involvement in *TRGC* rearrangement in human thymocytes. Expression of *RAG* genes, which mediate V(D)J recombination, was already evident in early CD34^+^ subsets in our bulk dataset (**Figure 5A**). In the single cell dataset, *RAG* expression was very low in the immature thymocyte stages and could not be reliably identified. However, transcription of *TRGC* and *TRDC* was clearly detected and can signify not only expression of a mature γ- or δ-chain but also ongoing rearrangement at these loci (**Figure 5B**). Notably, we observed that cells initially express *TRGC1* and later switch to *TRGC2*, while mature γδ T cells with surface expression of the γδ TCR almost exclusively use *TRGC2* (**Figure S4A**). This suggests that *TRGC2* is involved in the formation of the functional TCR, whereas *TRGC1* might only be transcribed in the course of rearrangement. We found that, according to pseudotime, *TCF3* and *TCF4* expression reached high levels at the same time as *TRGC1* and slightly before *TRGC2*, suggesting that they could be involved in coordinating chromatin opening and transcription of this region. We did indeed detect multiple TCF3 motifs at the *TCRG* locus, all of which were associated with regions of accessible chromatin and permissive histone marks in CD34^+^ thymocytes, indicative of an active role of TCF3 at these sites (**Figure 5C**). Some TCF4 and few TCF12 motifs were also observed, but these did not consistently align with any observable epigenetic features. Analysis of a potential relationship between expression of the E protein encoding genes and transcription of the *TCRG* locus revealed a positive correlation (r = 0.31) between *TCF3* and *TRGC2* levels in DN thymocytes, which was not observed to the same extent for *TCF4* or *TCF12* (r = -0.06 and r = 0.11, respectively) (**Figure 5D**). Curiously, this correlation was not detected for *TCF3* and *TRGC1* (r = -0.04), which seems to be driven by a subpopulation of immature DN thymocytes that express high levels of *TRGC1* but not *TCF3* (**Figure 5E**). This suggests that, in human, TCF3 may not be required to promote accessibility at the *TCRG* locus but instead might control the expression of the rearranged γ-chain to enable γδ TCR assembly. Despite similar expression windows of *TRDC* and *TRGC2*, no direct correlation was observed between *TCF3* and *TRDC* transcription (r = -0.03) (**Figure S4B**), which indicates that TCF3 is probably not responsible for controlling chromatin accessibility or active transcription at the TCRD locus. However, multiple TCF3 motifs were detected across the TCRD locus (**Figure S4C**), suggesting that TCF3 might be involved in coordinating V(D)J recombination of the δ-chain, as reported previously for TCF3 knockout mouse model (19, 82). Of note, TCF4 and TCF12 transcription was negatively or not at all correlated with that of TRDC and TRGC1 (**Figure S4D**) and few motifs were detected at either locus, therefore the two factors are unlikely to be key regulators of V(D)J recombination of TCRD and TCRG.

**Figure 6:**
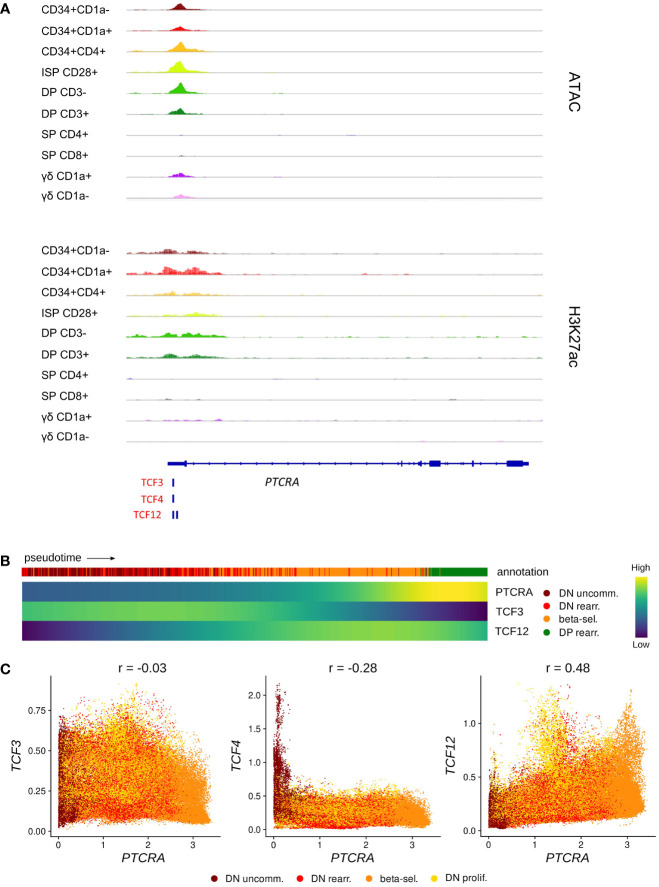
TCF12 is predicted to act as positive regulator of *PTCRA* transcription. **(A)** Genome browser view of ATAC, H3K27ac and E protein motifs in the *PTCRA* promoter region. Locations of promoters (P) and enhancers (E) were queried in the Ensembl Regulatory Build but none were found for this locus. **(B)** Heatmap showing gene expression along differentiation pseudotime in immature thymocytes. Smoothed gene expression was determined based on a generalized additive model fitted on the cell pseudotimes, cells in pseudotime window of interest were selected and expression was scaled by gene prior to visualization. **(C)** Scatter plot for imputed transcript levels of E protein encoding genes and *PTCRA* in immature thymocytes. Cells are colored by cluster and Pearson correlation coefficient is shown.

In human, rearrangement of the *TCRB* locus is thought to occur slightly after the *TCRD* and *TCRG* loci (36) and functionality of the β-chain is assessed by assembly with the surrogate pTα (encoded by *PTCRA*) to form the pre-TCR. TCF3 and TCF12 have both been shown to bind to regulatory sequences at the *Ptcra* locus in mouse thymocytes, but it seems that TCF3 is the main driver of *Ptcra* expression, whereas TCF12 plays a secondary synergistic role but is not able to induce high *Ptcra* transcription by itself (83). Using footprint analysis of our ATAC-seq data, we indeed identified a motif common for all E proteins as well as a TCF12-specific motif at the transcription start site of *PTCRA*, which overlapped with open chromatin and permissive H3K27 acetylation in immature thymocytes (**Figure 6A**). Gene expression analysis along pseudotime revealed that *PTCRA* transcription coincided with the upregulation of *TCF12* but was preceded by a *TCF3* expression peak (**Figure 6B**). Regulatory network prediction with SCENIC and SIGNET identified *PTCRA* as a potential target of TCF12, whereas the putative regulatory interaction between TCF3 and PTCRA was found to be weaker (SCENIC) or not detected at all (SIGNET). A possible role of TCF12 in *PTCRA* transcription was also supported by the finding that expression levels of both genes in DN thymocytes exhibit a positive correlation (r = 0.48), whereas no correlation was observed for *TCF3*/*TCF4* vs. *PTCRA* (r = -0.03 and r = -0.28, respectively) (**Figure 6C**). Together, these observations indicate that, similar to descriptions in mouse, E proteins might be involved in the transcriptional induction of *PTCRA* during human T cell development in the thymus. While the presented analyses seem to favor TCF12 rather than TCF3 as the main transcriptional regulator, *in vitro* validation will be required to assess the true impact of both E proteins on pTα expression and to explore any potential synergism or interdependence.

In summary, these observations imply that E proteins play a role in the indispensable processes that allow human thymocytes to develop into either αβ or γδ T cells, prior to the actual fate decision.

### 3.4 E and ID protein encoding genes in αβ T cell development

**Figure 7:**
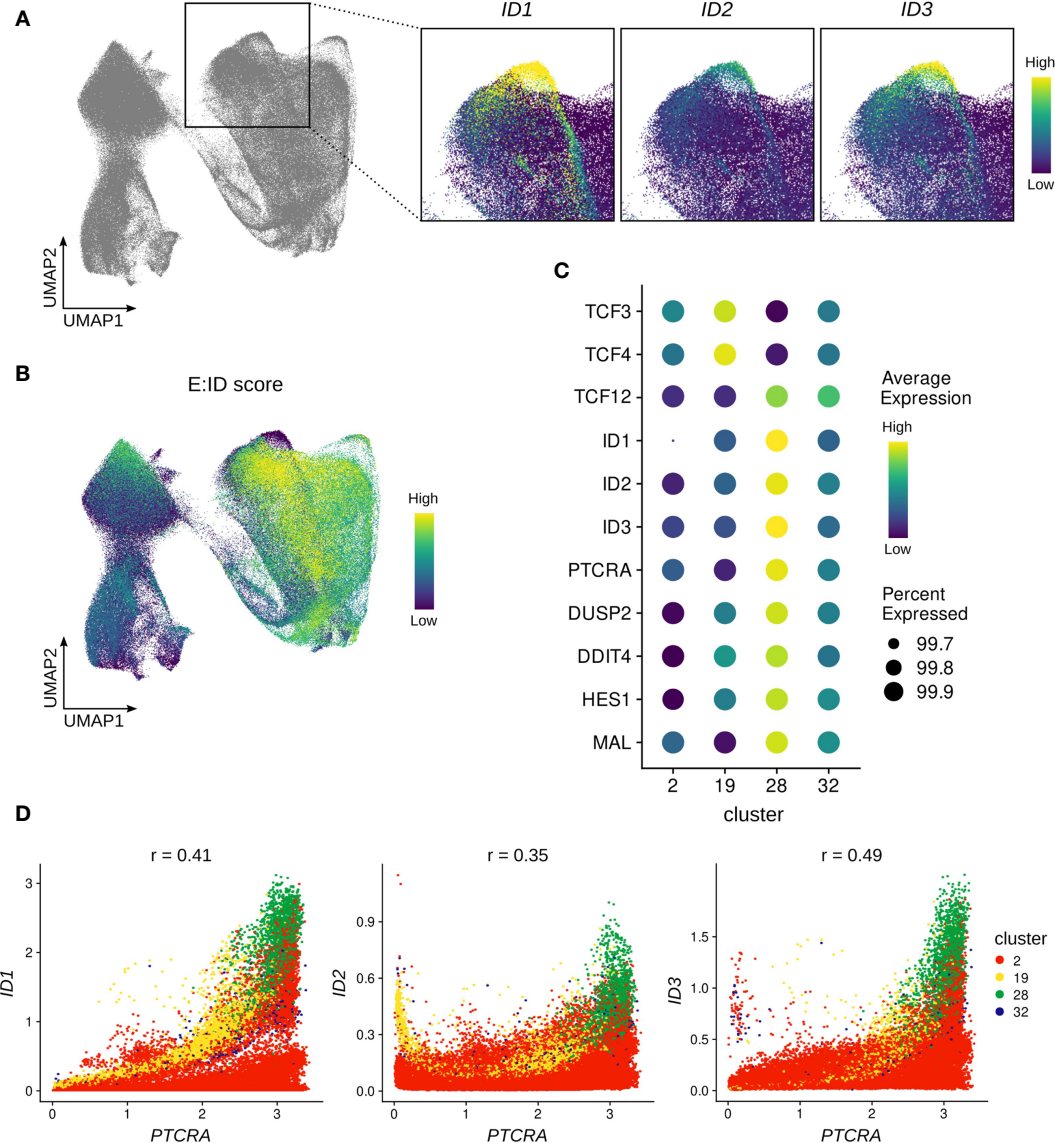
ID protein encoding genes are strongly induced in a subset of β-selecting thymocytes. **(A)** UMAP visualization of ID protein encoding gene expression in the β-selecting thymocyte cluster. **(B)** UMAP visualization of the E:ID score calculated on a per-cell basis. **(C)** Dot plot visualizing pseudo-bulk expression of E and ID protein encoding genes and cluster 28 marker genes. Imputed, gene-scaled expression is shown for all subclusters comprising the beta-selecting cluster. **(D)** Scatter plot for imputed transcript levels of ID protein encoding genes and *PTCRA* in β-selecting thymocytes. Cells are colored by β-selecting subcluster and Pearson correlation coefficient is shown.

A critical test that thymocytes need to pass on their path to become mature αβ T cells is β-selection, which involves assembly of the pre-TCR to assess the successful rearrangement of the TCR β-chain. Analysis of the single cell thymocyte data revealed a small subset of cells (cluster 28, **Figure S1C** and **Figure S5A**) within the β-selecting cluster that expressed unusually high levels of ID protein encoding genes (**Figure 7A**). This was particularly remarkable for *ID2* and *ID3*, for which widespread expression is only induced much later in development, as described above (**Figures 3B, C**). Since ID proteins are known to inhibit E proteins and high ID levels therefore indicate low E protein activity, we used gene signature scoring to determine an E:ID score based on E (positive weight) and ID (negative weight) transcript levels for each cell. Visualization on the UMAP confirmed an extremely low E:ID score for cluster 28, whereas surrounding cells exhibited a high score (**Figure 7B**). This indicates a rapid but temporally restricted transcriptional induction of ID protein encoding genes and suggests a high potential for robust E protein inhibition in this subset of cells. It has previously been demonstrated that E protein activity needs to be transiently shut down following β-selection to initiate differentiation of αβ-lineage thymocytes (25). Therefore, it is likely that cluster 28 reflects cells at this specific stage of human T cell development.

To further characterize cluster 28, we conducted differential gene expression analysis which identified *ID1*, *ID3*, and *PTCRA* as the main markers of this cluster. However, we also detected significantly elevated transcript levels for *DUSP2*, *DDIT4*, *HES1* and *MAL* in comparison to the rest of the dataset (**Figure 7C**). All of these genes have previously been linked to TCR signaling (84–87), and are therefore indicative of strong ongoing pre-TCR activity in cluster 28. It is known that *Id3* expression in thymocytes can be triggered *via* MAPK signaling as a consequence of TCR engagement (27), and it is possible that *ID1* and *ID2* can be similarly induced by pre-TCR signaling. Gene co-expression analysis did indeed reveal a positive correlation between *PTCRA* and *ID1* (r=0.41) or *ID3* (r=0.49) transcript levels in cells of the β-selection cluster, with cluster 28 cells exhibiting the highest expression levels for all 3 genes (**Figure 7D**). Moreover, GRN analysis predicted a regulatory connection between ID1 and PTCRA, although the nature and direction of the relationship cannot easily be established for non-transcription factors using this approach.

It has been proposed that the relatively weak signal transmitted by the pre-TCR is insufficient to permit further maturation of αβ-lineage cells, and that supplementary Notch signaling is required to achieve transient E protein inhibition and thereby developmental progression (88). NOTCH gene signature scoring, based on expression of known NOTCH target genes (see Material & Methods), did indeed show a high score in cluster 28, providing an explanation for the high levels of the NOTCH target *HES1* in these cells. Nevertheless, the score was equally high in the remaining cells in the β-selection cluster and therefore cannot fully explain the isolated upregulation of ID protein encoding genes (**Figure S5B**). Finally, to rule out a potential contamination with cells expressing a γδ TCR as a source of strong TCR signaling, we assessed *TRGC2*/*TRDC* transcription in the cells from cluster 28 which confirmed substantially lower levels compared to the γδ T cell subclusters (**Figure S5C**). This strongly suggests that pre-TCR signaling can induce high levels of ID gene expression in human thymocytes in a subset of β-selecting cells.

**Figure 8:**
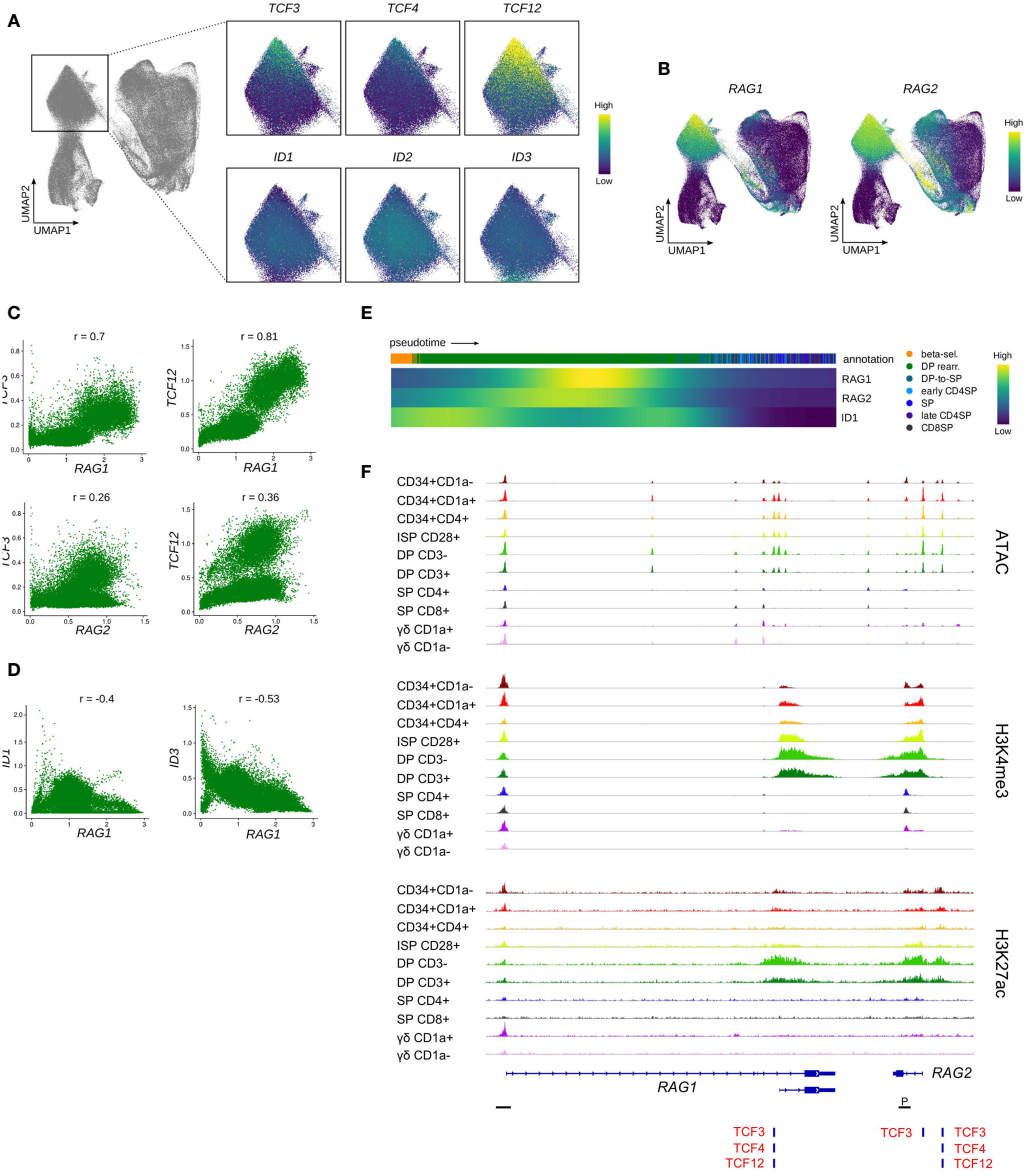
Transcription of E protein encoding genes is associated with *RAG* expression in rearranging DP thymocytes. **(A)** UMAP visualization of E and ID protein encoding gene expression in the rearranging DP thymocyte cluster. **(B)** UMAP visualization of *RAG1* and *RAG2* expression according to the single cell thymocyte data set. Transcripts were imputed prior to visualization. **(C)** Scatter plot for imputed transcript levels of *TCF3*, *TCF12*, *RAG1* and *RAG2* in rearranging DP thymocytes. Pearson correlation coefficient is shown. **(D)** Scatter plot for imputed transcript levels of *ID1*, *ID3*, and *RAG1* in rearranging DP thymocytes. Pearson correlation coefficient is shown. **(E)** Heatmap showing gene expression along differentiation pseudotime in DP and SP thymocytes. Smoothed gene expression was determined based on a generalized additive model fitted on the cell pseudotimes, cells in pseudotime window of interest were selected and expression was scaled by gene prior to visualization. **(F)** Genome browser view of ATAC, H3K4me3, H3K27ac and E protein motifs at regulatory regions of the *RAG* gene locus. Locations of promoters (P) were retrieved from the Ensembl Regulatory Build and are indicated below the gene structure.

Following β-selection, thymocytes progress to the DP stage which encompasses the rearrangement of the *TCRA* locus. Assessment of the rearranging DP cluster in the single cell data indicated a gradual decrease in the transcript levels of all three E proteins with highest levels observed in the most immature rearranging DPs and low levels in cells that started to embark on the transition to the SP stage (**Figure 8A**). Incidentally, the subgroup of cells with elevated transcription of E protein encoding genes exhibited relatively low ID gene expression. This was also clearly demonstrated by the previously determined E:ID score which indicated that cells undergo a rapid switch from a high to low E-ID ratio as they mature (**Figure 7B**). Analysis of *RAG* expression levels revealed high *RAG1* and *RAG2* quantities in the (E:ID)^high^ cell group (**Figure 8B**). In line with this, gene-gene co-expression analysis confirmed a positive correlation between *TCF3*/*TCF12* and *RAG1*/*2* transcript levels (**Figure 8C**), whereas *ID1*/*ID3* levels were anticorrelated with those of *RAG1* (**Figure 8D**). Expression profiles along pseudotime also pointed towards an inverse expression pattern for RAG genes and *ID1* (**Figure 8E**). Regulon prediction suggested TCF12 and ID1 as putative regulators of *RAG1*/*2*, but no regulatory relationship with TCF3 was detected. However, a TCF3 binding site was indeed identified at the transcription start site of *RAG2*, in addition to consensus E protein binding sites at a putative upstream enhancer and at the transcription start site of the short *RAG1* isoform, which were all associated with increased accessibility and a permissive epigenetic signature in DP thymocytes (**Figure 8F**). Together, these findings suggest a possible role for TCF12 and potentially also TCF3 in the upregulation of *RAG* genes in DP thymocytes. Binding of TCF12 and TCF3 to the *Rag* locus has indeed been shown before, and *Tcf3*- or *Tcf3*/*Tcf12*-deficient mice display a moderate or severe impairment in the upregulation of *Rag1* and *Rag2* in DP thymocytes (89, 90). Hence, our observation in human thymocytes is in line with previously published mouse data describing crucial roles of E proteins during *Tcra* rearrangement *via* regulation of Rag expression. Of note, the role of TCF12 in DP thymocytes seems to extend further to regulation of cell viability *via* transcriptional upregulation of *Rorc*. We could also confirm a positive correlation for *TCF12* and *RORC* expression in our data (r=0.73) (**Figure S6A**) and a regulatory relationship between the two factors was identified *via* GRN analysis.

In contrast, the role of ID1 in rearranging DP thymocytes has not been studied in much detail, but some reports suggest that *Id1* overexpression during murine T and B cell development results in severely reduced *Rag1*/*2* expression (91, 92). In addition, it is known that *Id3* needs to be downregulated in DP thymocytes to permit *Rag* expression (93) and *Id3* overexpression in thymocytes results in reduced *Rag1*/*2* levels (94). This indicates that *ID1* and *ID3* expression in DP thymocytes negatively regulates *RAG* transcription and therefore modulates or terminates *TCRA* rearrangement. Given the staggered timepoints of upregulation, it is likely that ID1 only has a moderate effect on *RAG* transcription, whereas *ID3* induction coincides with and might therefore be responsible for the complete shutdown of *RAG* expression (**Figure 8B** and **Figure 3C**). Since this takes place around the positive/negative selection stage as indicated by the upregulation of *TRAC* and *CD5* (**Figure S6B**), initiation of *ID3* expression may represent a response to TCR signaling and subsequent downregulation of *RAG1/2* would be required to prohibit further rearrangements in positively selected cells.

In summary, our data support the hypothesis that, like in mouse, TCF3 and TCF12 are involved in the upregulation of *RAG* expression in rearranging DP human thymocytes, whereas ID1 and ID3 seem to exert an inhibitory function towards *RAG* transcription. Whether this is achieved solely through E protein inhibition or involves other regulatory mechanisms remains to be explored.

### 3.5 E and ID protein encoding genes in non-conventional T cells

**Figure 9:**
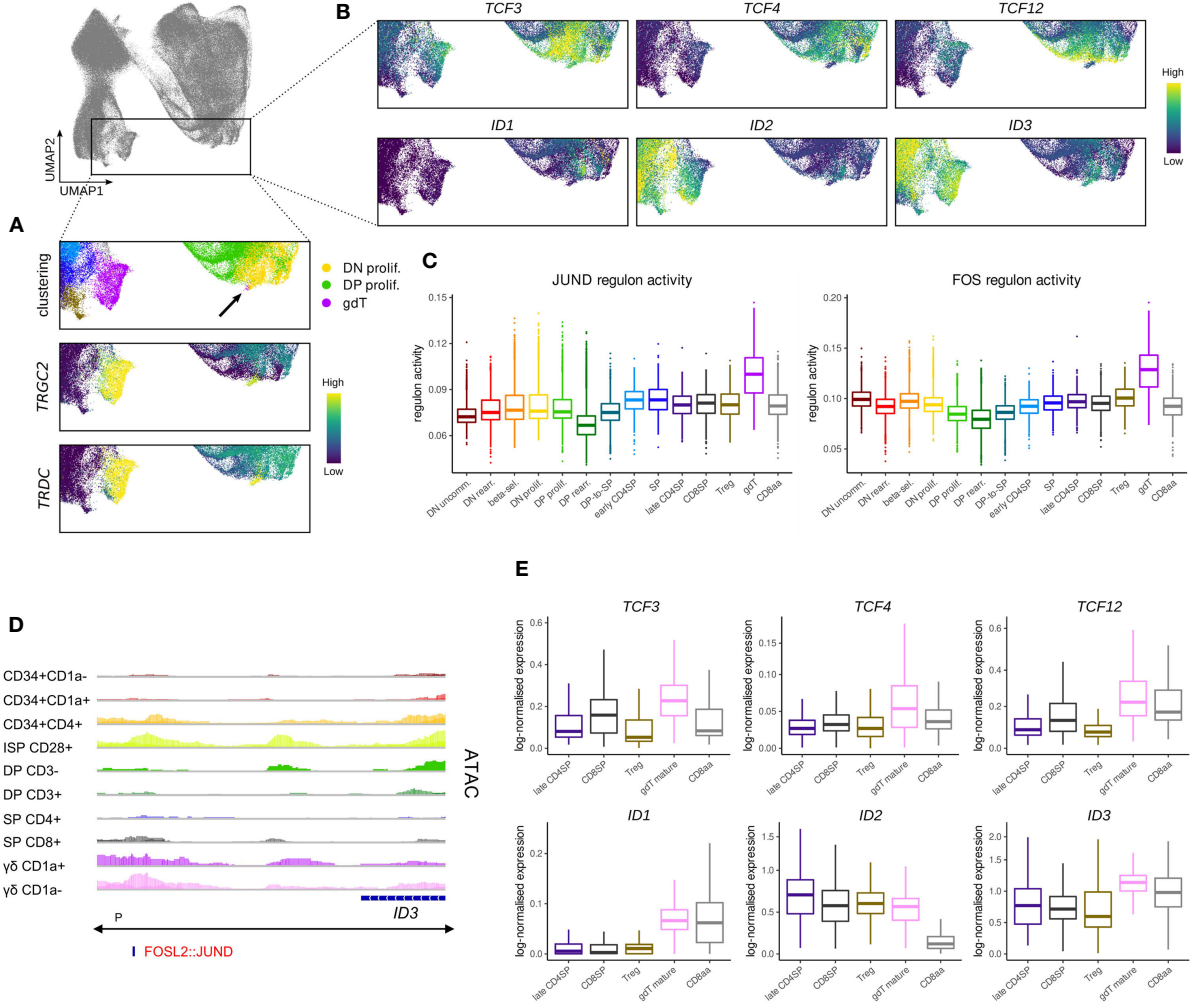
Transcription of *TCF4* and *TCF12* but not *TCF3* is shut down in cells committing to the γδ lineage. **(A)** UMAP visualization of *TRGC2* and *TRDC* expression in thymocytes of the γδ lineage. Annotated clusters are depicted in the top panel. **(B)** UMAP visualization of E and ID protein encoding genes in thymocytes of the γδ lineage. **(C)** Activity of JUND and FOS regulons per cluster as predicted by running pySCENIC on the single cell data set. **(D)** Genome browser view of chromatin accessibility and AP-1 family motif at a regulatory region downstream of *ID3*. Location of the ID3 promoter (P), as indicated below the gene structure, was found to span the entire locus. **(E)** Pseudo-bulk expression of E and ID protein encoding genes in mature thymocytes based on the single cell data set. For γδ lineage cells only a subcluster of mature γδTCR^+^ cells as indicated by low levels of CD1a was included.

The inclusion of γδ T cells and CD8αα T cells in our established datasets allowed us to assess expression of E and ID protein encoding genes in these non-conventional T cell types. ID3 is a well-known regulator of γδ T cell development and has been shown to be upregulated following strong TCR signaling. Consistent with this and the understanding that strong TCR signals are associated with adoption of γδ fate, *ID3* levels were highest in immature γδ T cells according to bulk RNA-seq analyses (**Figure 3C**). In the single cell data, we identified a subset of cells with surface γδ TCR expression that displayed a γδ-lineage gene expression signature according to clustering results but that still grouped with DN thymocytes (**Figure 9A**). Moreover, this subset of cells showed very low expression of maturation markers such as CD73 (encoded by *NT5E*), *CD44*, *CD27*, *CD69* and *IL7R* and γδ effector genes, like *NKG7*, *KLRB1* and *GNLY*, were not yet upregulated (**Figure S6C**), which identifies them as very immature γδ T cells. Curiously, *ID3* levels were only moderate in this subset (**Figure 9B**), which suggests that these cells have only just received a TCR signal and are still in the process of upregulating *ID3*.

Regulon prediction in the single cell dataset identified two regulons for the AP-1 family transcription factors FOS and JUND that displayed especially strong activity in γδ T cells (**Figure 9C**). *ID3* was suggested as a target gene of both regulons and in support of that, we were able to identify an AP-1 family motif downstream of the *ID3* gene at differentially accessible sites in γδ T cells (**Figure 9D**), indicating that the two factors might indeed confer *ID3* upregulation. Importantly, AP-1 transcription factors are known downstream mediators of TCR signaling, which validates induction of *ID3* transcription as a result of TCR activity.

Further analysis of the immature γδ T populations in bulk and single cell data suggested that these cells do not express notable levels of *TCF4*, *TCF12*, *ID1* or *ID2* at this stage (**Figure 9B**). However, we noted high levels of *TCF3* in the very immature subset which only dropped gradually as γδ T cells became more mature. While TCF3 is known to play a crucial role in *Tcrg*/*Tcrd* locus gene rearrangement (72, 81), and is therefore indispensable for γδ T cell development, additional roles in γδ-lineage differentiation processes have not been studied in much detail. It is possible that TCF3 protein activity is quickly diminished following *ID3* induction since ID3 has been shown to not only inhibit TCF3 function but also to mediate a reduction in protein levels (88). Nevertheless, it remains unclear why *TCF3* transcripts continue to be expressed following γδ-lineage commitment whereas *TCF12* expression is extinguished much more rapidly.

Comparison of transcript levels for E and ID protein encoding genes in the mature cell types that we identified in the single cell data confirmed generally low expression of *TCF3*, *TCF4* and *TCF12* in conventional αβ-lineage cells as well as in γδ and CD8αα^+^ T cells, with some minor variability between cell types (**Figure 9E**). *ID1* levels appeared to be higher in γδ and CD8αα^+^ T cells, but due to the low total transcript quantities, the biological relevance of this difference may be negligible. In contrast, *ID2* levels were remarkably similar in the γδ and conventional αβ lineage cells, while much lower quantities were detected in CD8αα^+^ cells (**Figure 9E**). This contradicts findings in murine CD8αα^+^ T cells, which appear to exhibit higher *Id2* levels in comparison with CD8αβ^+^ T cells (95, 96). It is possible that this difference stems from the analysis of thymic vs. peripheral cells. However, it has been proposed that CD8αα^+^ T cell development is independent of ID2 (97), in which case the biological significance of differential *ID2* transcription is uncertain. *ID3* levels were only slightly higher in mature γδ T cells compared to the other analyzed cell types. This could be explained by the moderate downregulation of *ID3* that is associated with the maturation of γδ T cells and the upregulation of *ID3* in SP thymocytes. ID3 is often described as γδ-specific transcriptional modulator, but these observations suggest that this characteristic only extends to immature cell types, perhaps reflecting TCR signaling events that impact the lineage choice.

Notably, the regulatory T cells that were identified in the single cell dataset expressed similar levels of all E and ID genes as CD4^+^ and CD8^+^ SPs, which indicates that mature naïve αβ T cells do not exhibit differential transcription of these factors.

### 3.6 E and ID protein encoding genes during prenatal T cell development

T cell differentiation in the thymus starts very early during embryonic development and especially cells of the γδ lineage have been shown to exhibit notable differences between prenatal and postnatal origin, although this has been predominantly studied in mice thus far. To investigate potential changes in E and ID gene expression that are associated with human development, we used published single cell data (64, 98, 99) to establish a prenatal dataset consisting of around 112.000 cells from 20 donors, with samples covering a continuous age window from 8 weeks post conception (wpc) to 17 wpc (**Figure S7A, Table S1**). To visualize the age progression while retaining enough cells from each cell type to avoid donor- or source-specific biases, we further distinguished between cells from embryonic (≤ 10 wpc) and fetal (>10 wpc) donors (**Figure S7B, C**). We used the pediatric dataset as reference for automated cell type annotation *via* label transfer to identify clusters with similar gene expression profiles (**Figures S7C-E**).

**Figure 10:**
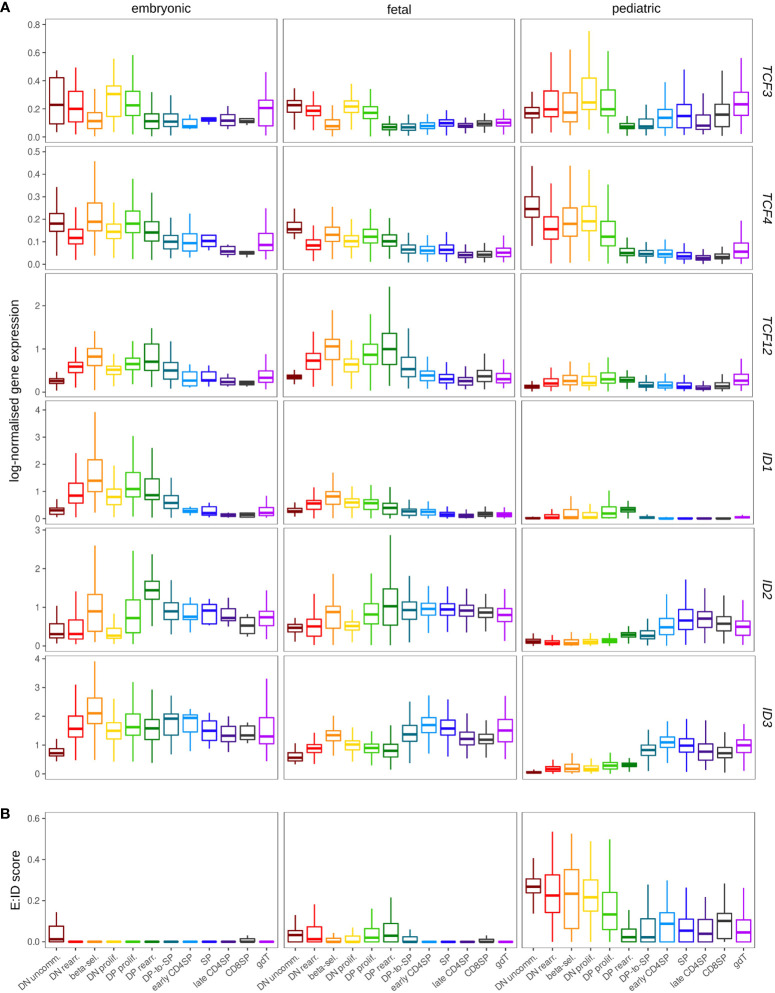
Expression of ID protein encoding genes exhibits different patterns in the pre- and postnatal thymocytes. **(A)** Pseudo-bulk expression of E and ID protein encoding genes in thymocytes based on prenatal and pediatric single cell data sets. The prenatal data was divided into embryonic (≤10 wpc) and fetal (>10 wpc) samples. Note that only very few SP, early/late CD4^+^ and CD8^+^ SP thymocytes were detected in embryonic samples (see **Figure S7C**). **(B)** Average E:ID score per cell type in embryonic, fetal, and pediatric thymocytes.

Comparison of embryonic, fetal and pediatric data revealed substantially higher transcript levels for *ID1* and *TCF12* in prenatal thymocytes (**Figure 10A**). Elevated *ID1* levels in fetal thymocytes have indeed been described before in mice (41), but the biological significance remains unclear. Strikingly, gene expression profiles for *ID2* and *ID3* differed substantially between prenatal and postnatal thymocytes. Whereas both genes were only upregulated at the DP-SP transition in pediatric T cell development as laid out above, their induction was shifted to much earlier stages in prenatal development (**Figure 10A** and **Figure S7F**). This is remarkable because it signifies substantial levels of ID gene expression in DN thymocytes, which we generally determined to be an ID^low^ phase in the pediatric thymus. The anticipated consequence of this is a more pronounced repression of E protein activity in immature prenatal thymocytes, which is also supported by the difference in the E:ID score in pre- and postnatal samples (**Figure 10B**). This might directly influence thymocyte maturation and differentiation based on the roles of TCF3 and TCF12 described above. Hence, it is possible that elevated *TCF12* levels in embryonic and fetal thymocytes represent a compensatory mechanism to retain some TCF12 activity despite strong ID gene expression.

## 4 Discussion

In this manuscript, we have established an overview of the regulation of E and ID protein encoding genes during human T cell development, using both bulk and single cell profiling methods to understand gene expression and epigenetic regulation of these genes and their regulatory networks. Comparison with murine thymocytes revealed some potential differences in the stage-specific expression and thus most likely also the activity of these genes. Furthermore, a remarkable shift in the E/ID gene expression ratio was observed in the early stages of human T cell development during the transition from fetal to postnatal thymopoiesis.

Our analyses revealed several interesting differences and similarities between the gene expression dynamics of E and ID protein encoding genes. Both *ID1* and *TCF12* seem to be largely absent in immature and mature thymocytes and instead reach their expression peak when cells are midway through their developmental progression towards αβ-lineage cells. In contrast, *TCF3* and *TCF4* expression levels are highest in immature stages and extinguished in mature thymocytes, whereas *ID2* and *ID3* display the opposite pattern with upregulation relatively late in the developmental course. However, the expression windows for *TCF3*/*TCF4* and *ID2*/*ID3* are not completely identical, which suggests that their regulation is controlled by different upstream mechanisms. Importantly, the inverse transcription pattern for E and ID proteins indicates a crucial requirement for E protein expression in the early phase of T cell development but possibly also a need for E protein shutdown *via* degradation or inhibitory dimerization with ID proteins at later stages.

At the epigenetic level, the promoter histone modification H3K4me3 was highly correlated with RNA expression for all E and ID protein encoding genes, as anticipated. Remarkably, however, the corresponding chromatin regions remained largely accessible, and thus permissive for expression, throughout all developmental stages, rendering it feasible to rapidly alter the expression levels in response to new regulatory inputs that can be derived from both environmental and intracellular stimuli. This is important in the case of E and ID proteins given their strong involvement in both TCR generation and signaling, respectively, which are both critical determinators of thymocyte maturation.

Some of the E and ID protein encoding gene expression patterns displayed remarkable features. According to the single cell data, *TCF3* RNA levels were found to be highest in proliferating DN and DP thymocytes, which seems to contradict previous reports of TCF3 acting as inhibitor of proliferation in support of TCR rearrangements, to which E proteins also contribute by regulating *RAG* gene expression. This may point towards differences between RNA and protein levels as well as additional layers of protein activity regulation. E proteins not only form heterodimers with ID proteins that inhibit their activity, they also heterodimerize with other tissue and stage specific factors that thereby can regulate E protein activity (100). In addition, it is established that E protein phosphorylation can induce degradation, for instance following ERK activation downstream of NOTCH and TCR signaling (26, 29).

A surprising characteristic that we observed was the heterogeneous *ID1* expression in β-selecting and rearranging DNs that partially overlapped with *ID3* expression in those early stages. While the *ID1* expression in the rearranging DNs may reflect some early thymocytes that have just successfully rearranged the TCR β-chain and thus are on their way to go through the β-selection process, the difference in *ID1* and *ID3* expression in β-selected cells is intriguing and we hypothesize that this may possibly reflect a differential impact of both ID proteins with respect to their impact on E protein dependent RAG expression or TCR gene locus accessibility. Such differential mechanisms following β-selection may relate to the preferential usage of the distal versus proximal TCRα V gene segments during the development of CD8αα versus the conventional CD8αβ T cells, which has previously been observed (101). Indeed, that biased use of V-J pairs in CD8αα T cells appears to deviate between the pre- and postnatal thymus (64), in line with the developmental differences in *ID1* levels that we detected. In addition, it has been shown in mice that TCF3 is involved in controlling the order of *Tcrg* rearrangements and thereby determines which γδ TCR clonotypes can be generated (19, 42). Clonotypes that are exclusively generated before birth seem to make use of *Tcrg* elements that do not rely on TCF3 presence for their recombination. In contrast, TCF3 activity is required during postnatal γδ T cell development to prevent rearrangement of said fetal-specific region and instead permit a switch to different clonotypes. Although it is unclear if there are preferential ID/E protein dimerization complexes, ID1 and ID3 may have similar differential impacts on *TCRA* V gene segment usage following β-selection. Similarly, we speculate that the high ID levels we observed in the prenatal DN thymocytes may control TCF3 activity in order to ensure correctly timed *TCRG* locus rearrangement, which may lead to the development of fetal γδ T cells with restricted TCR diversity (39). In any case, it has previously been shown in mice that αβ lineage development is not disrupted in absence of ID3 (29). Thus, the simultaneous upregulation of *ID1*, *ID2* and *ID3* observed in our dataset indicates a possible compensation by the other family members in *Id3*-deficient thymocytes. Whether or not ID1 and ID2 might have a specific role following human β-selection remains to be investigated, but our analysis clearly points towards a fast and strong but also highly transient upregulation of ID gene expression in response to pre-TCR signaling, most likely to achieve temporary inhibition of E protein activity to prevent further TCR rearrangements during this proliferative transition.

In the course of investigating a potential role of *TCF3* in the rearrangement of the *TCRG* locus in postnatal development, we noted that mature γδ T cells exhibited strong preferential usage of *TRGC2*, whereas both *TRGC1* and *TRGC2* were actively transcribed in DN thymocytes. While there is evidence that in other mammals *Trgc* usage can differ between thymus and periphery and that circulating γδ T cells vary in their expression of different *Trgc* segments (102, 103), we hypothesize that our observation is instead related to the age-dependent generation of distinct γδ subsets. It has previously been shown that *TRGC1* is predominantly used by Vγ9Vδ2^+^ cells, while *TRGC2* does not display preferential association with certain *TRGV* segments (104). Vγ9Vδ2^+^ cells are mainly generated in early fetal development and a switch to Vδ2^–^ subtypes takes place in mid-gestation (105). As a consequence, Vγ9Vδ2^+^ cells only make up a small minority of γδ T cells in the postnatal thymus, which provides an explanation for the low expression of *TRGC1* in mature thymic γδ T cells observed in our pediatric data set. In line with this, *TRGC1* expression in DN thymocytes is likely caused by germline transcription at the *TCRG* locus but might not reflect any actual involvement in the assembly of a functional γ-chain. Assessment of *TRGC1* and *TRGC2* transcript levels in γδ T cells identified in the prenatal data set did not reveal a bias for either segment, which seems to confirm that preferential *TRGC2* usage is an age-specific phenomenon. Due to the limited number of γδ T cells in the prenatal data set and batch effects between samples from different developmental stages, a more detailed investigation of the potential shift from *TRGC1* to *TRGC2* was not possible. Targeted enrichment of γδ T cells from fetal thymi in combination with TCR sequencing will be key to further unravel the use of different TRGC segments in pre- and postnatally developing γδ T cells.

Although the expression patterns for *TCF3* and *TCF12* seem to point towards similar preferential requirements for γδ and αβ T cell development, respectively, as observed in mice (8, 106), both the bulk and single cell RNA-seq expression profiles do reveal stages of overlapping expression which may relate to both redundant and/or unique regulatory roles with respect to TCR rearrangements or other processes that control T cell development. Combined with the largely overlapping *ID1*/*ID3* and *ID2*/*ID3* expression patterns during early and late human T cell development, respectively, and in the absence of any solid information on E-ID dimerization preferences, it is clear that functional studies with genetic approaches will be required to fully understand the specific roles of the E and ID proteins during human T cell development. Given the altered expression ratio of E/ID protein encoding genes during pre- and postnatal human T cell development, this will be required in both developmental windows and should be feasible now using CRISPR-mediated gene-editing tools in combination with the available *in vitro* models that support human T-lineage differentiation from various stem cell and hematopoietic precursor sources (16, 107, 108).

In summary, we here provide an in-depth analysis of the transcriptional dynamics of E and ID protein encoding genes in human postnatal thymocytes and provide insights into how these integrate in the broader molecular mechanisms that control distinct stages of human T cell development, both upstream and downstream of these genes. Our study provides novel insights into the unique regulatory roles of E and ID proteins during human T cell development and encourages additional research to unravel their detailed function in this context.

## Data availability statement

The newly generated data presented in the study are deposited in the National Center for Biotechnology Information Gene Expression Omnibus (GEO repository, accession code GSE205439. Previously generated and published pediatric data from our lab are available on GEO with the accession codes GSE151081 (46), GSE144870 (63) and GSE206710 (64) and on ArrayExpress with the accession code E-MTAB-8581 (64). Publicly available single cell datasets were retrieved from GEO with the accession code GSE139042 (65). Normalized gene count tables for mouse bulk RNA-seq data were obtained from the ImmGen dataset repository (http://rstats.immgen.org/DataPage/, GEO accession GSE109125). The publicly available prenatal single cell datasets were retrieved from ArrayExpress (accession code E-MTAB-8581) (64), GEO (accession code GSE133341) (99) and NODE (accession code OEP001185) (98).

## Ethics statement

The studies involving human participants were reviewed and approved by Ghent University Hospital ethical committee. Written informed consent to participate in this study was provided by the participants’ legal guardian/next of kin.

## Author contributions

Study conception and design: LB, JR, TT. Data collection: AK, LB, ML, SS, FVN. Analysis and interpretation of results: LB, JR, JVH, TP, GL, BV, TT. Draft manuscript preparation: LB, JR, JVH, TT. All authors reviewed the results and approved the final version of the manuscript.

## Funding

This work was supported by the Fund for Scientific Research Flanders (FWO, grants G053816N and G075421N, fellowship to SS), The Concerted Research Action from the Ghent University Research Fund (GOA, BOF18-GOA-024), The Chan Zuckerberg Initiative (CZF2019-002445), The Foundation against Cancer (Stichting Tegen Kanker, grant 2020-084) and the Cancer Research Institute Ghent (CRIG, YIPOC grant). The computational resources and services used in this work were provided by the VSC (Flemish Supercomputer Center), funded by the FWO and the Flemish Government – department EWI.

## Acknowledgments

We thank the flow cytometry core facility from the UGent Faculty of Medicine and Health Sciences, K. Francois and G. Van Nooten (Department of Human Structure and Repair, Ghent University Hospital) for thymus tissue, Ellen De Meester from NXTGNT and Niels Vandamme from the VIB single cell core for help with the single cell work.

## Conflict of interest

The authors declare that the research was conducted in the absence of any commercial or financial relationships that could be construed as a potential conflict of interest.

## Publisher’s note

All claims expressed in this article are solely those of the authors and do not necessarily represent those of their affiliated organizations, or those of the publisher, the editors and the reviewers. Any product that may be evaluated in this article, or claim that may be made by its manufacturer, is not guaranteed or endorsed by the publisher.
