# Supplementary material for: Transcriptional dynamics and epigenetic regulation of E and ID protein encoding genes during human T cell development
Source: Front Immunol. 2022 Jul 28;13:960918. doi: 10.3389/fimmu.2022.960918 (PMC9366357; doi:10.3389/fimmu.2022.960918)
Supplement: Supplementary file 1 [file DataSheet_1.pdf]

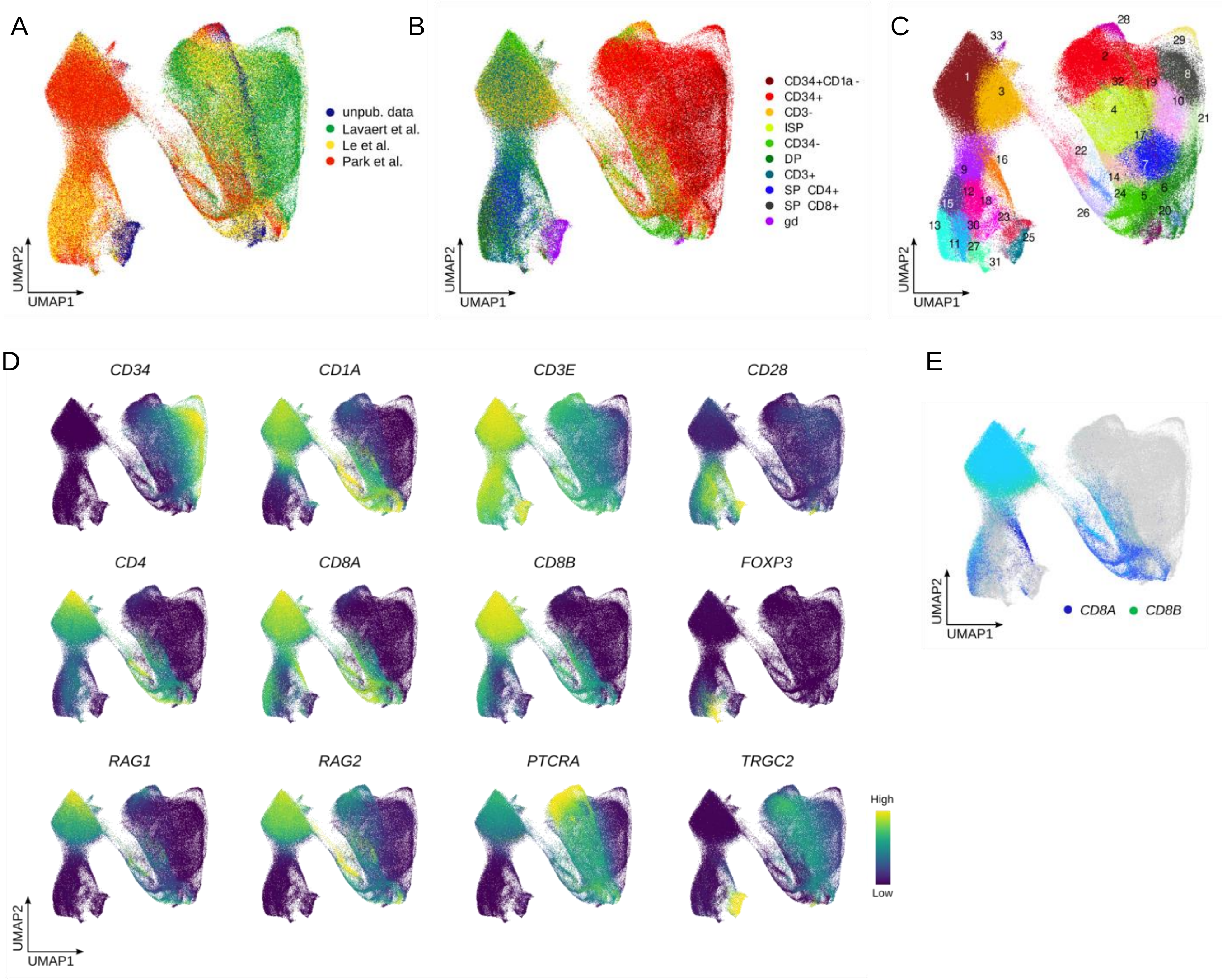

Figure S1: Composition of the pediatric single cell data set.

(A) UMAP visualization of thymocytes in the pediatric single cell data set by source. Note that the respective studies used enrichment methods to obtain different thymocytes subsets. (B) UMAP visualization of thymocytes in the pediatric single cell data set by enrichment strategy. (C) UMAP visualization of initially identified clusters in the pediatric single cell data set. Clusters with comparable expression of thymocyte marker genes were merged to form the larger annotated clusters used throughout the study (see Figure 1C). (D) UMAP visualizations of imputed expression of known thymocyte marker genes in the pediatric single cell data set. (E) UMAP visualization of transcription of *CD8A* vs. *CD8B* used to identify CD8αα^{+} thymocytes.

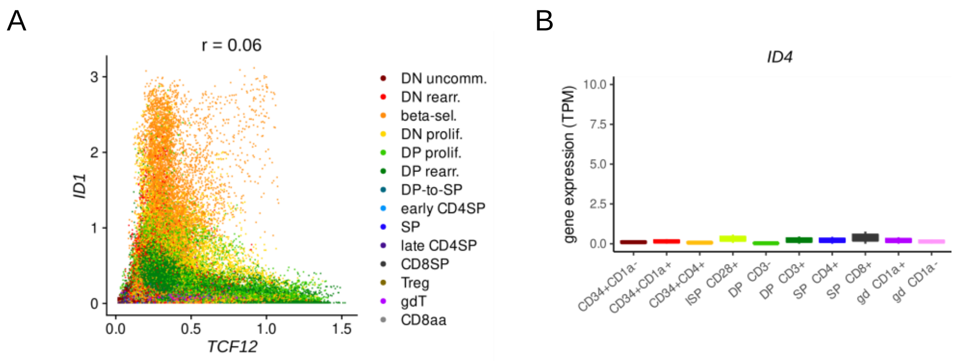

Figure S2: ***ID4*** **is not expressed in human thymocytes.**

(A) Scatter plot for imputed transcript levels of *ID1* and *TCF12* in thymocytes. Cells are colored by celltype and Pearson correlation coefficient is shown. (B) Transcript levels of *ID4* in human thymocytes according to bulk RNA-seq data.

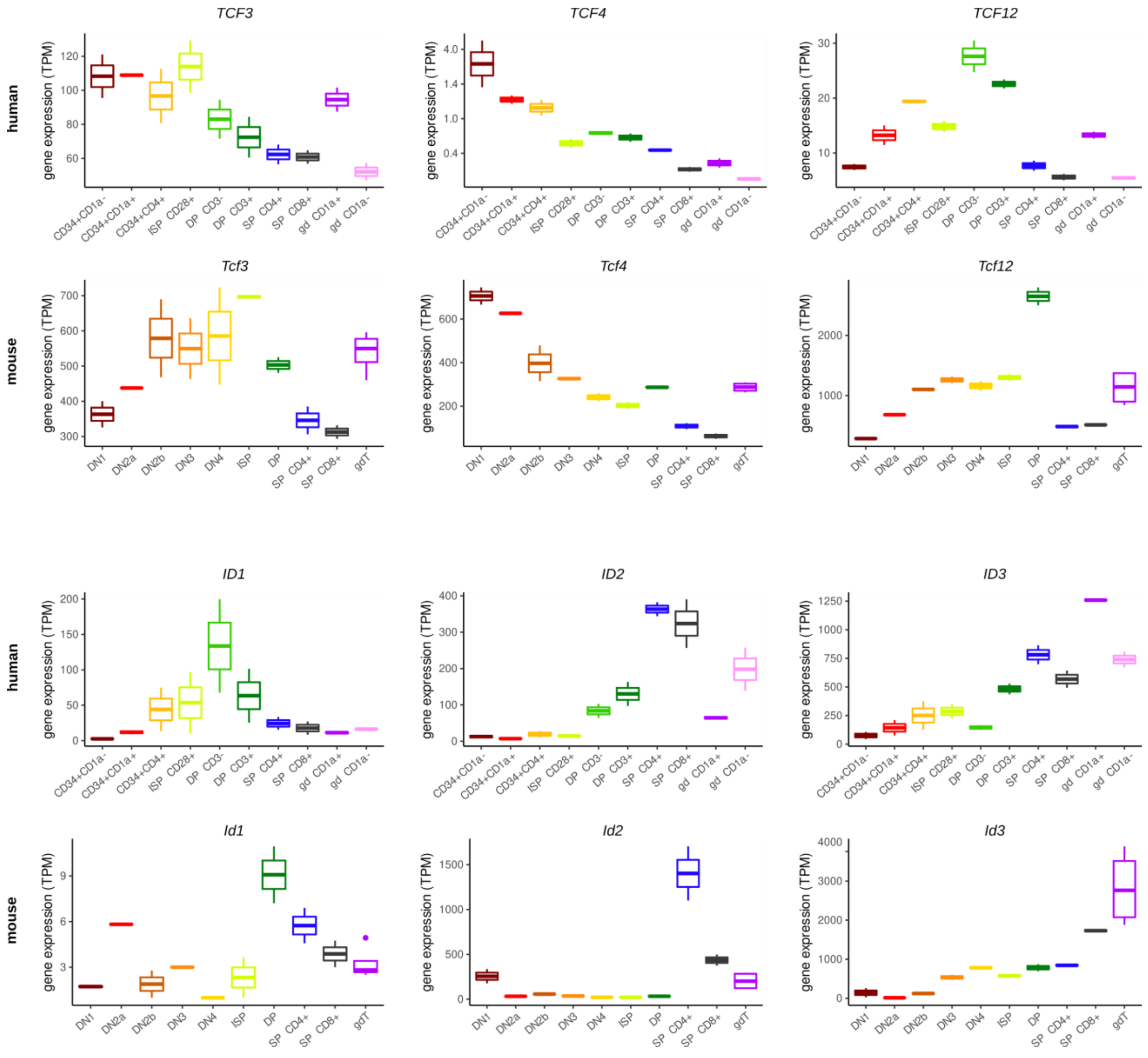

Figure S3: E and ID protein encoding genes exhibit similar expression patters in human and murine thymocytes.

Transcript levels of E and ID protein genes according to bulk RNA-seq on human and mouse thymocytes. Mouse data was obtained from the ImmGen dataset repository as a normalized count table.

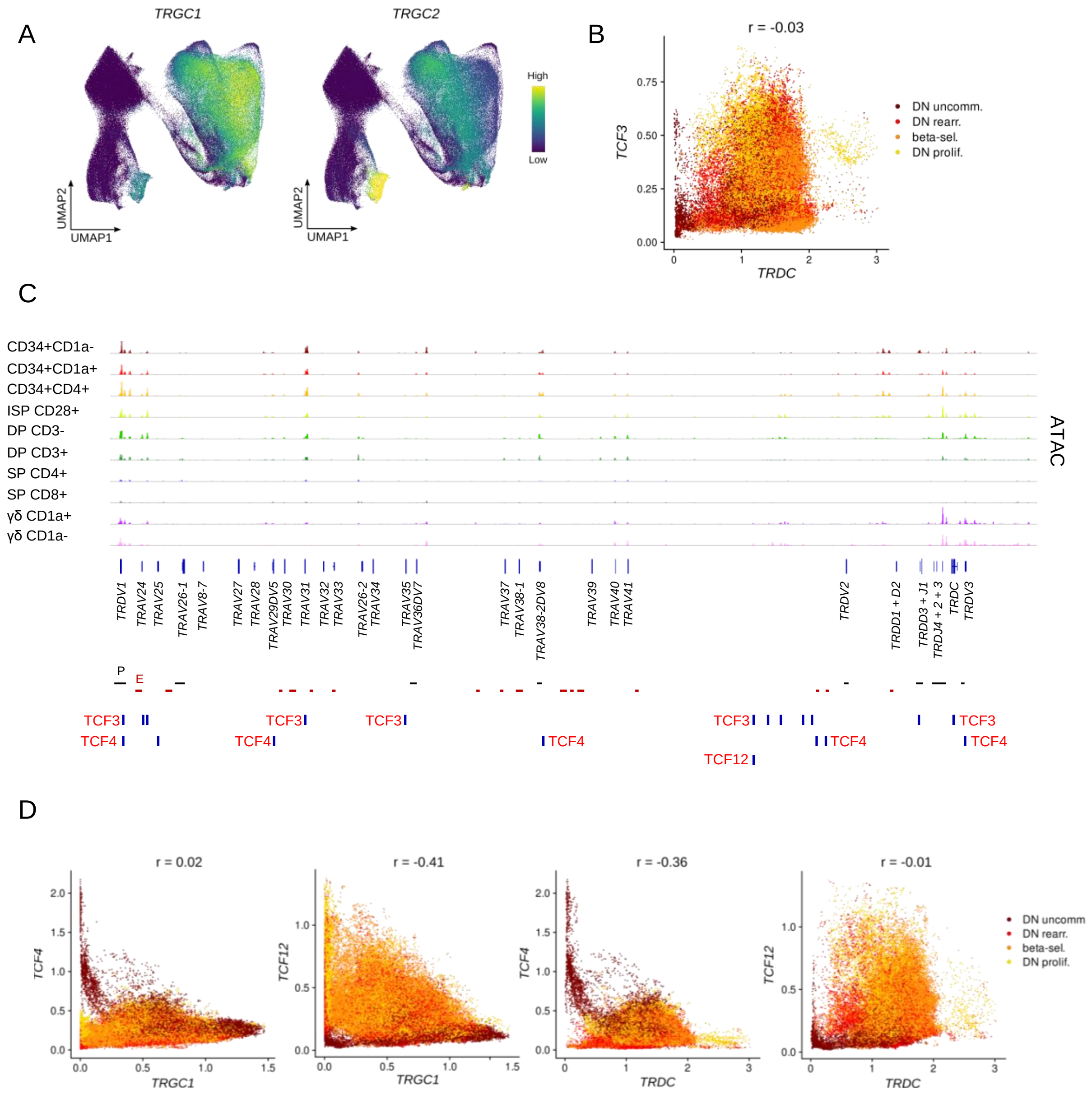

Figure S4: *TRGC* expression in human thymocytes.

(A) UMAP visualization of imputed *TRGC1* and *TRGC2* transcripts in pediatric thymocytes. (B) Scatter plot for imputed transcript levels of *TCF3* and *TRDC* in immature thymocytes. Cells are colored by celltype and Pearson correlation coefficient is shown. (C) Genome browser view of ATAC and E protein motifs at the *TCRD* locus. Locations of promotors (P) and enhancers (E) were retrieved from the Ensembl Regulatory Build and are indicated below in the gene structure. (D) Scatter plots for imputed transcript levels of *TCF4*, *TCF12*, *TRGC1* and *TRDC* in immature thymocytes. Cells are colored by celltype and Pearson correlation coefficient is shown.

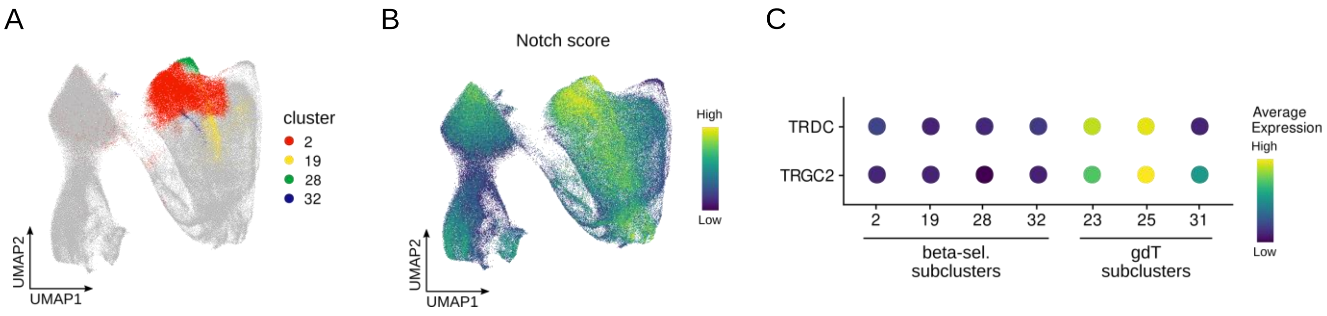

Figure S5: Subclusters of β-selecting thymocytes.

(A) UMAP visualization of originally identified clusters that were merged to form the β-selecting cluster based on similar expression of key thymocyte marker genes. (B) UMAP visualization of the Notch score determined based on the expression of known NOTCH signaling target genes. (C) Dot plot visualizing pseudo-bulk expression of *TRDC* and *TRGC2*. Imputed, gene-scaled expression is shown for all subclusters comprising the β-selecting and γδ T clusters.

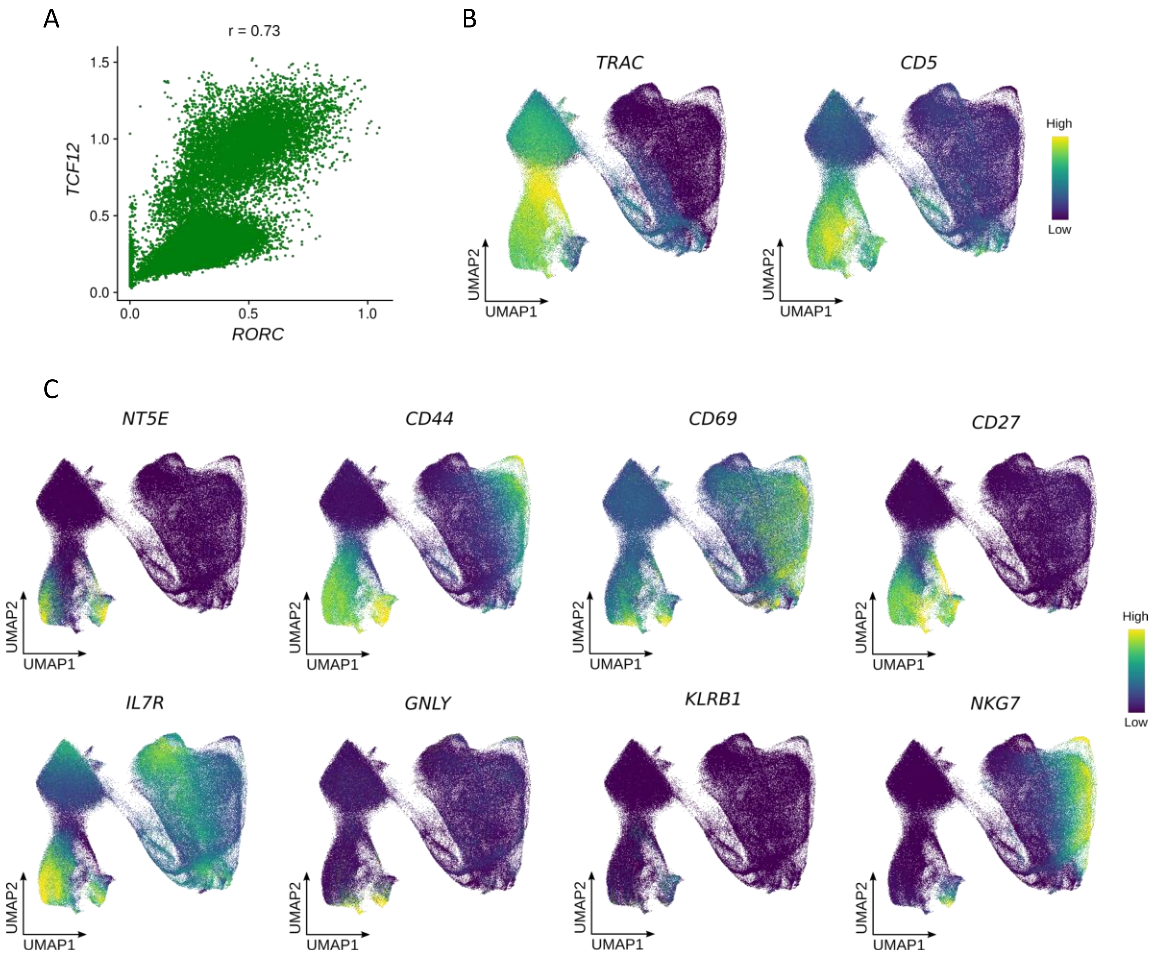

Figure S6: Expression of *TCF12* and *RORC* is strongly correlated in rearranging DP thymocytes.

(A) Scatter plot for imputed transcript levels of *TCF12* and *RORC* in rearranging DP thymocytes. Pearson correlation coefficient is shown. (B) UMAP visualization of imputed *TRAC* and *CD5* expression in pediatric thymocytes. (C) UMAP visualizations of imputed expression of known γδ T cell maturation genes in the pediatric single cell data set.

Figure S7

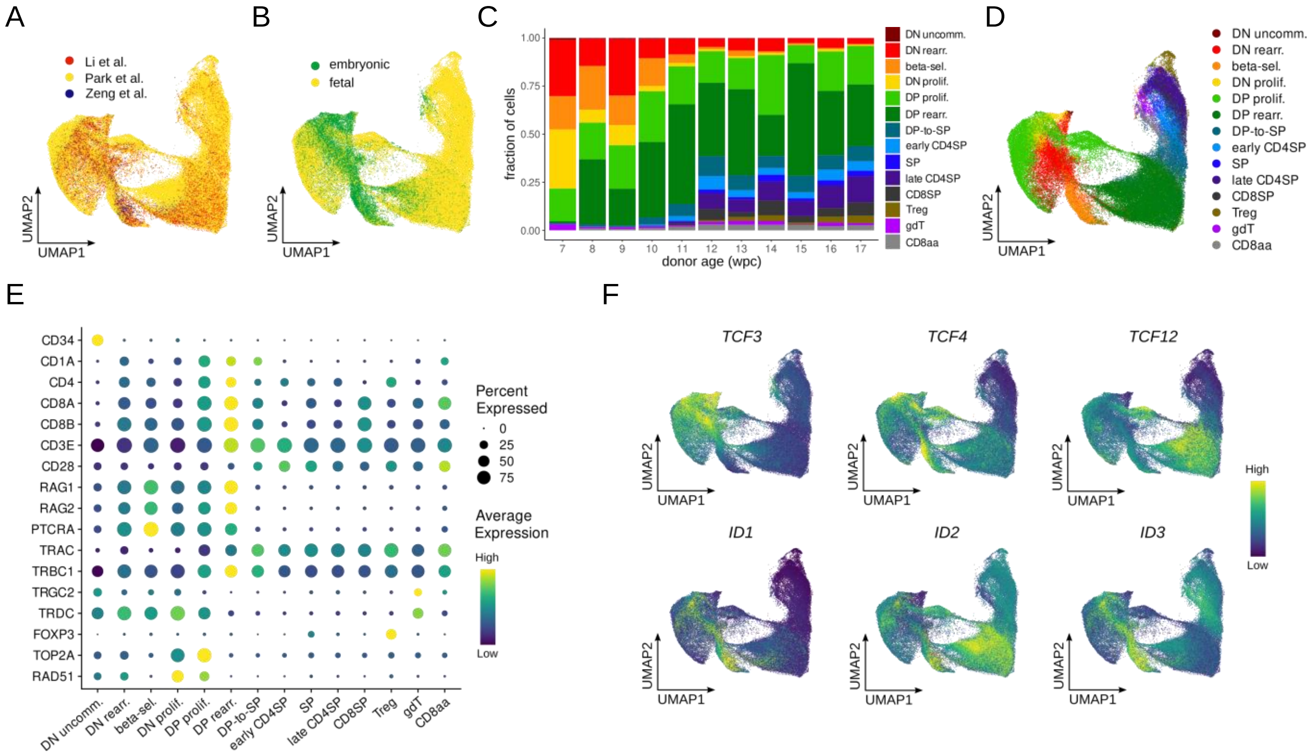

Figure S7: Composition of the prenatal single cell data set.

(A) UMAP visualization of thymocytes in the prenatal single cell data set by source. (B) UMAP visualization of thymocytes in the prenatal single cell data set by developmental stage. Embryonic: ≤10 wpc, fetal: >10 wpc. (C) Stacked bargraph indicating the proportion of celltypes detected by age. (D) UMAP visualizing the annotated clusters in the prenatal single cell thymus data set after automated annotation based on the pediatric reference data. (E) Dot plot visualizing pseudo-bulk expression of known thymocyte markers per annotated cluster in the prenatal single cell data set. Non-imputed data was log-normalized, averaged and scaled by gene. (F) UMAP visualizing the imputed expression of E and ID protein encoding genes in the prenatal thymus.

Table S1: Overview of single cell libraries used in this paper.

Unique identifier	Source	Donor	Age	Dev. stage	Sex	Enrichment strategy	Database	Accession info
5478STDY7935100	Park et al.	Park_1	9 wpc	embryonic	f	total cells	ArrayExpress	E-MTAB-8581
FCAImmP7179369	Park et al.	Park_2	16 wpc	fetal	m	CD45pos	ArrayExpress	E-MTAB-8581
FCAImmP7198432	Park et al.	Park_3	9 wpc	embryonic	f	total cells	ArrayExpress	E-MTAB-8581
FCAImmP7198634	Park et al.	Park_4	11 wpc	fetal	m	CD45pos	ArrayExpress	E-MTAB-8581
FCAImmP7277556	Park et al.	Park_5	17 wpc	fetal	f	CD45pos	ArrayExpress	E-MTAB-8581
FCAImmP7277564	Park et al.	Park_6	14 wpc	fetal	m	CD45pos	ArrayExpress	E-MTAB-8581
FCAImmP7292030	Park et al.	Park_5	17 wpc	fetal	f	CD45pos	ArrayExpress	E-MTAB-8581
FCAImmP7292034	Park et al.	Park_6	14 wpc	fetal	m	CD45pos	ArrayExpress	E-MTAB-8581
FCAImmP7528283	Park et al.	Park_7	13 wpc	fetal	m	CD45pos	ArrayExpress	E-MTAB-8581
FCAImmP7528294	Park et al.	Park_7	13 wpc	fetal	m	CD45pos	ArrayExpress	E-MTAB-8581
FCAImmP7555851	Park et al.	Park_8	16 wpc	fetal	f	CD45pos	ArrayExpress	E-MTAB-8581
FCAImmP7555860	Park et al.	Park_8	16 wpc	fetal	f	CD45pos	ArrayExpress	E-MTAB-8581
FCAImmP7579218	Park et al.	Park_9	12 wpc	fetal	f	CD45pos	ArrayExpress	E-MTAB-8581
FCAImmP7579230	Park et al.	Park_9	12 wpc	fetal	f	CD45pos	ArrayExpress	E-MTAB-8581
FCAImmP7851890	Park et al.	Park_10	11 wpc	fetal	m	total cells	ArrayExpress	E-MTAB-8581
FCAImmP7851891	Park et al.	Park_10	11 wpc	fetal	m	total cells	ArrayExpress	E-MTAB-8581
FCAImmP7851892	Park et al.	Park_11	12 wpc	fetal	f	total cells	ArrayExpress	E-MTAB-8581
FCAImmP7851893	Park et al.	Park_11	12 wpc	fetal	f	total cells	ArrayExpress	E-MTAB-8581
FCAImmP7851894	Park et al.	Park_11	12 wpc	fetal	f	total cells	ArrayExpress	E-MTAB-8581
FCAImmP7851895	Park et al.	Park_11	12 wpc	fetal	f	total cells	ArrayExpress	E-MTAB-8581
WSSS8084742	Park et al.	Park_12	7 wpc	embryonic	f	total cells	ArrayExpress	E-MTAB-8581
WSSS8084743	Park et al.	Park_12	7 wpc	embryonic	f	total cells	ArrayExpress	E-MTAB-8581
WSSS8084744	Park et al.	Park_13	8 wpc	embryonic	m	total cells	ArrayExpress	E-MTAB-8581
WSSS8084745	Park et al.	Park_13	8 wpc	embryonic	m	total cells	ArrayExpress	E-MTAB-8581
GSM3906003	Zeng et al.	Zeng_1	8 wpc	embryonic	f	CD235aneg	GEO	GSE133341
GSM3906005	Zeng et al.	Zeng_2	9 wpc	embryonic	m	CD235aneg	GEO	GSE133341
GSM3906004	Zeng et al.	Zeng_3	10 wpc	embryonic	m	CD235aneg	GEO	GSE133341
OER087534	Li et al.	Li_1	11 wpc	fetal	m	7-AADneg	NODE	OEP001185
OER087535	Li et al.	Li_2	15 wpc	fetal	m	7-AADneg	NODE	OEP001185
OER087536	Li et al.	Li_3	13 wpc	fetal	m	7-AADneg	NODE	OEP001185
OER087537	Li et al.	Li_4	9 wpc	embryonic	m	7-AADneg	NODE	OEP001185
LMB1	This paper	Boehme_1	4 m	pediatric	m	DP	GEO	GSE205439
LMB2	This paper	Boehme_1	4 m	pediatric	m	CD34pos	GEO	GSE205439
LMB3	This paper	Boehme_1	4 m	pediatric	m	gd	GEO	GSE205439
LMB4	This paper	Boehme_1	4 m	pediatric	m	ISP	GEO	GSE205439
SRR10302342	Le et al.	Le_1	1 y 7m	pediatric	m	CD34pos	GEO	GSE139042
SRR10302343	Le et al.	Le_1	1 y 7m	pediatric	m	CD34pos	GEO	GSE139042
SRR10302344	Le et al.	Le_1	1 y 7m	pediatric	m	CD34neg	GEO	GSE139042
SRR10302345	Le et al.	Le_1	1 y 7m	pediatric	m	CD34neg	GEO	GSE139042
SRR11680329	Le et al.	Le_2	1 y 11m	pediatric	m	CD34pos	GEO	GSE139042
SRR11680330	Le et al.	Le_2	1 y 11m	pediatric	m	CD34pos	GEO	GSE139042
SRR11680331	Le et al.	Le_2	1 y 11m	pediatric	m	CD34pos	GEO	GSE139042
SRR11680332	Le et al.	Le_2	1 y 11m	pediatric	m	CD34pos	GEO	GSE139042
SRR11680333	Le et al.	Le_3	9 d	pediatric	m	CD34pos	GEO	GSE139042
SRR11680334	Le et al.	Le_3	9 d	pediatric	m	CD34pos	GEO	GSE139042
SRR11680335	Le et al.	Le_3	9 d	pediatric	m	CD34pos	GEO	GSE139042
SRR11680336	Le et al.	Le_3	9 d	pediatric	m	CD34neg	GEO	GSE139042
SRR11680337	Le et al.	Le_3	9 d	pediatric	m	CD34neg	GEO	GSE139042
SRR11680338	Le et al.	Le_3	9 d	pediatric	m	CD34neg	GEO	GSE139042
SRR11680345	Le et al.	Le_4	5 y	pediatric	f	CD34neg	GEO	GSE139042
SRR11680346	Le et al.	Le_4	5 y	pediatric	f	CD34neg	GEO	GSE139042
SRR11680347	Le et al.	Le_4	5 y	pediatric	f	CD34neg	GEO	GSE139042
SRR11680348	Le et al.	Le_4	5 y	pediatric	f	CD4SP	GEO	GSE139042
SRR11680349	Le et al.	Le_4	5 y	pediatric	f	CD4SP	GEO	GSE139042
SRR11680350	Le et al.	Le_4	5 y	pediatric	f	CD4SP	GEO	GSE139042
SRR11680351	Le et al.	Le_4	5 y	pediatric	f	CD8SP	GEO	GSE139042
SRR11680352	Le et al.	Le_4	5 y	pediatric	f	CD8SP	GEO	GSE139042
SRR11680353	Le et al.	Le_4	5 y	pediatric	f	CD8SP	GEO	GSE139042
S1	Lavaert et al.	Lavaert_1	5 m	pediatric	f	CD34posCD1Aneg	GEO	GSE144870
S2	Lavaert et al.	Lavaert_2	5 m	pediatric	m	CD34posCD1Aneg	GEO	GSE144870
S3	Lavaert et al.	Lavaert_3	6 m	pediatric	f	CD34posCD1Aneg	GEO	GSE144870
CISE10	Lavaert et al.	Lavaert_1	5 m	pediatric	f	CD34pos	GEO	GSE144870
CISE11	Lavaert et al.	Lavaert_2	5 m	pediatric	m	CD34pos	GEO	GSE144870
TTA15	Lavaert et al.	Lavaert_4	1 y 2 m	pediatric	m	CD34pos	GEO	GSE144870
TTA16	Lavaert et al.	Lavaert_5	9 m	pediatric	m	CD34pos	GEO	GSE144870
TTA7	Park et al.	Park_14	1 y 2 m	pediatric	m	CD3neg	GEO	GSE206710
TTA8	Park et al.	Park_14	1 y 2 m	pediatric	m	CD3neg	GEO	GSE206710
TTA9	Park et al.	Park_14	1 y 2 m	pediatric	m	CD3pos	GEO	GSE206710
TTA10	Park et al.	Park_14	1 y 2 m	pediatric	m	CD3pos	GEO	GSE206710
TTA11	Park et al.	Park_15	13 y 4 m	pediatric	m	CD3neg	GEO	GSE206710
TTA12	Park et al.	Park_15	13 y 4 m	pediatric	m	CD3pos	GEO	GSE206710
TTA13	Park et al.	Park_16	6 m	pediatric	m	CD3neg	GEO	GSE206710
TTA14	Park et al.	Park_16	6 m	pediatric	m	CD3pos	GEO	GSE206710
